# Single-cell nucleic acid profiling in droplets (SNAPD) enables high-throughput analysis of heterogeneous cell populations

**DOI:** 10.1093/nar/gkab577

**Published:** 2021-07-07

**Authors:** Leland B Hyman, Clare R Christopher, Philip A Romero

**Affiliations:** Graduate Program in Cell and Molecular Biology, University of Wisconsin–Madison, Madison, WI 53706, USA; Department of Biochemistry, University of Wisconsin–Madison, Madison, WI 53706, USA; Department of Biochemistry, University of Wisconsin–Madison, Madison, WI 53706, USA; Department of Biochemistry, University of Wisconsin–Madison, Madison, WI 53706, USA; Department of Chemical & Biological Engineering, University of Wisconsin–Madison, Madison, WI 53706, USA; The University of Wisconsin Carbone Cancer Center, Madison, WI 53706, USA

## Abstract

Experimental methods that capture the individual properties of single cells are revealing the key role of cell-to-cell variability in countless biological processes. These single-cell methods are becoming increasingly important across the life sciences in fields such as immunology, regenerative medicine and cancer biology. In addition to high-dimensional transcriptomic techniques such as single-cell RNA sequencing, there is a need for fast, simple and high-throughput assays to enumerate cell samples based on RNA biomarkers. In this work, we present single-cell nucleic acid profiling in droplets (SNAPD) to analyze sets of transcriptional markers in tens of thousands of single mammalian cells. Individual cells are encapsulated in aqueous droplets on a microfluidic chip and the RNA markers in each cell are amplified. Molecular logic circuits then integrate these amplicons to categorize cells based on the transcriptional markers and produce a detectable fluorescence output. SNAPD is capable of analyzing over 100,000 cells per hour and can be used to quantify distinct cell types within heterogeneous populations, detect rare cells at frequencies down to 0.1% and enrich specific cell types using microfluidic sorting. SNAPD provides a simple, rapid, low cost and scalable approach to study complex phenotypes in heterogeneous cell populations.

## INTRODUCTION

The complex phenotypes displayed by multicellular organisms arise from interactions between highly specialized cell subpopulations. This intercellular heterogeneity plays a pivotal role in physiological (1,2), developmental (3,4) and disease processes (5–7). Transcriptomic analyses have traditionally been performed on the bulk collection of cells within a biological sample, and thus report the average transcriptional state of the population. This average does not capture the individuality of single cells within the population (8). Single-cell transcriptomic approaches such as single-cell RNA sequencing (scRNA-seq (9–11)) are helping to elucidate the role of cellular heterogeneity in fields ranging from immunology (12,13) to oncology (5–7). These methods have revealed the continuum of individual cells states during differentiation processes such as hematopoiesis (14) and secondary tumor formation (15). They have also been used to characterize cellular heterogeneity in tumor micro-environments, where small subpopulations of cancer stem cells can drive disease progression and resistance to treatment (16). Single-cell transcriptomic approaches are essential for studying biological processes that involve phenotypically heterogeneous cell populations.

While scRNA-seq is a powerful method that provides rich transcriptomic data for thousands of cells, experiments generally carry high expense and long turnaround times. There are many cases where it may be more advantageous to quickly and inexpensively enumerate cell samples based on a smaller set of RNA biomarkers. Flow cytometry-based fluorescent *insitu* hybridization (Flow-FISH (17)) and microfluidic PCR-activated cell sorting (PACS (18,19)) have each demonstrated success in these scenarios; however, they require laborious and involved sample processing that can span over multiple days. Thus, despite these recent technological advances, there is still need for new single cell transcriptional profiling methods that are low cost, high-throughput, simple and rapid.

Droplet-based microfluidics is a powerful means to analyze single cells in high-throughput (20–22). These systems encapsulate individual cells into picoliter-scale aqueous droplets, and each of these cells can be studied and analyzed in isolation from the bulk population. These techniques can achieve similar throughput to flow cytometry, but provide a more general format that can be adapted to a diverse array of biomolecular assays and readouts (23). For instance, droplet-based microfluidics has been used to perform single-cell reverse transcriptase-polymerase chain reaction (RT-PCR) to detect a specific mRNA transcript in tens of thousands of cells (18). While this approach is powerful, it suffers from a fundamental problem with droplet-based assays: cells encapsulated and lysed in droplets will often result in high lysate concentrations that inhibit downstream assays such as PCR (24). As a result, droplet-based PCR methods require complicated, multi-step workflows to remove reaction inhibitors, making assays difficult and less reliable. We therefore sought a simple droplet microfluidic method to detect mRNA biomarkers in hundreds of thousands of cells while avoiding PCR-based signal amplification altogether.

An ideal RNA amplification method for droplets could be performed in highly concentrated lysate with comparable sensitivity and selectivity to PCR. We identified reverse transcription-loop-mediated isothermal amplification (RT-LAMP) as a method that satisfies these criteria. LAMP is an isothermal nucleic acid amplification method that uses a strand displacing polymerase (*Bst* Polymerase) to generate dumbbell DNA structures and exponentially extend them to longer concatemers (25). Unlike DNA polymerases used in other methods, we found that *Bst* polymerase is resistant to lysate concentrations exceeding 10^6^ cells/mL (Supplementary Figure S1). LAMP-based assays have achieved limits of detection as low as 0.4 aM, which is comparable to nested PCR (26,27). These properties have facilitated LAMP-based digital nucleic acid quantification assays in microwells (28), SlipChips (29), centrifugal devices (30,31) and droplets (32–34). In previous work, others have adapted LAMP to analyze single cells through multi-step droplet workflows (35). However, we leveraged the lysate tolerance of LAMP to develop an unprecedentedly simple droplet-based single-cell analysis workflow which does not require any splitting, merging, or sorting steps.

In this work, we demonstrate a scalable and streamlined technique for profiling RNA biomarkers in tens of thousands of single mammalian cells. We take advantage of recent advances in droplet microfluidics (19,36) and molecular detection (37,38) to develop the novel SNAPD (single-cell nucleic acid profiling in droplets) platform that is capable of profiling several mRNAs simultaneously across tens of thousands of mammalian cells. Our method starts by encapsulating single cells into microdroplets, followed by isothermal amplification of target RNAs to produce a fluorescent signal. The resulting droplets can be analyzed directly to determine the proportion of a specific cell type within a heterogeneous cell mixture, or the droplets can be sorted to enrich a particular cell type and collect its DNA and RNA. Hundreds of droplets can be analyzed per second, allowing high-throughput analysis and sorting. SNAPD can be multiplexed to examine multiple RNAs simultaneously, and these multidimensional signals can be integrated via molecular computation to capture more complex cellular phenotypes. The SNAPD workflow is simpler and more streamlined than comparable methods such as PACS and Flow-FISH. Live cells are loaded directly into a microfluidic device with minimal processing beforehand and the entire experiment can be completed within a few hours. Due to its throughput, ease and low cost, SNAPD provides a scalable and facile solution for single-cell RNA biomarker analysis.

## MATERIALS AND METHODS

### LAMP primer design

For each gene target, we first used data from the Human Protein Atlas Project (39) to identify its most highly expressed transcriptional isoforms in our target cell type. We then used the Ensembl Genome Browser (40) to identify common regions between highly expressed isoforms, and designed LAMP primers to target these sequence regions. We designed LAMP primers using PrimerExplorer V5 software (Eiken Chemical Co., http://primerexplorer.jp/lampv5e). We found SNAPD’s sensitivity was greatly improved if we used two different sets of LAMP primers to target each transcript. In these cases, we designed primers such that little or no overlap occurred between them.

### DNA complexes and primer mixes

We ordered all DNA oligos from Integrated DNA Technologies (Coralville, IA, USA), and dissolved each into nuclease-free water (Thermo Fisher) prior to storage at −20°C. On the day of an experiment, we prepared stocks of each DNA complex in DEPC-treated phosphate-buffered saline (PBS) by slowly annealing the strands from 97°C to 23°C at a rate of −2°C/min. We then stored these DNA complexes on ice and protected from light until the time of experiment. We prepared LAMP primer mix stocks in nuclease-free water (Thermo Fisher) and stored at −20°C.

### Production of mRNAs using *in vitro* transcription

DNA templates for *KRT19*, *VIM* and *ERBB2* transcripts were synthesized by Integrated DNA Technologies (Coralville, IA, USA). We cloned these genes into the pET-22b vector under a T7 promoter. We performed *in vitro* transcription using the HiScribe™ T7 High Yield RNA Synthesis Kit (New England Biolabs), and purified the resulting RNA using a GeneJET RNA Purification Kit (Thermo Scientific). We quantified each RNA sample's concentration using a NanoDrop™ Spectrophotometer (Thermo Scientific) and stored RNA stocks at −80°C in DEPC-treated PBS.

### Microfluidic device fabrication

We fabricated microfluidic devices from polydimethylsiloxane (PDMS) via a soft photolithography process. We first deposited SU-8 3025 or 3010 photoresist onto a silicon wafer and spun to achieve the desired layer height. Next, we used a photomask to pattern microfluidic channels on the wafer and removed uncrosslinked regions with SU-8 developer. We then placed the patterned wafer into a petri dish, submerged in uncured PDMS (Dow Corning Sylgard^®^ 184) (11:1 polymer:cross-linker ratio) and cured at 72°C for at least 1 h. After PDMS polymerization, we excised the patterned PDMS device from the mold and bonded it to a glass microscope slide via plasma treatment. Finally, we treated the microfluidic channels with Aquapel (Pittsburgh Glass Works) to make the channels hydrophobic.

### Cell culture and staining

We subcultured MOLT-4 cells (American Type Culture Collection) in a 1:8 ratio every 2 days in RPMI-1640 medium (Gibco) supplemented with 10% fetal bovine serum (FBS) (Gibco) and 1X Antibiotic-Antimycotic (Gibco). We subcultured SK-BR3, MCF7 and U-2 OS cells (American Type Culture Collection) in a 1:4 ratio every 2 days and in Dulbecco's-modified Eagle's medium (DMEM), high glucose (Gibco) supplemented with 10% FBS and 1× Antibiotic-Antimycotic. On the day of experiment, we collected each cell type and washed twice with DPBS (Gibco). We then stained cells with 10 μM dye in DPBS for 30 min on ice. In droplet transducer and OR gate experiments, we stained with CellTrace™ Calcein AM (Invitrogen). In all other droplet experiments, we used CellTrace™ Calcein Red-Orange AM (Invitrogen). We then washed cells twice and resuspended in FluoroBrite™ DMEM (Gibco) containing 12.4 U/μl RNase I_f_ (New England Biolabs) and 0.025 U/μl DNase I (Thermo Fisher). We performed the *ESR1* experiment (Supplementary Figure S2), multiplex transducer experiment (Figure 3C and D) and droplet OR gate experiment (Figure 4G) with an earlier protocol that did not include nuclease treatment and used DPBS instead of DMEM.

### Bulk LAMP and DNA logic assays

We performed bulk RT-LAMP and DNA logic assays on purified RNAs, cells and mixtures of cells in triplicate using a Bio Rad CFX Connect quantitative PCR (qPCR) machine. We incubated the reactions at 65°C and monitored FAM, HEX and/or SYBR fluorescence channels. Each reaction comprised a total volume of 10 μl, consisting of 1.6 μM each FIP/BIP primer, 0.2 μM each F3/B3 Primer, 0.4 μM each LoopF/B Primer, 1× WarmStart LAMP Master Mix (New England Biolabs) and 0.5 U/μl SUPERase•In™ RNase Inhibitor (Invitrogen). We added DNA complexes at varying concentrations given in Supplementary Table S2. LAMP primer and logic gate sequences are shown in Supplementary Table S1. In reactions without any DNA complexes, we included LAMP Fluorescent Dye (New England Biolabs) as a general LAMP indicator. For experiments performed on purified RNAs, we added *in vitro* transcribed *KRT19, VIM* and/or *PTPRC* RNAs at the concentrations shown in Supplementary Table S2. For experiments performed on cells, we also included 2.5% Tween-20 (Sigma Aldrich) in the reaction buffer to act as a lysis reagent. We then added intact, unstained cells immediately before starting an experiment at a final concentration of 50 cells/μl per cell type.

### Single-cell experiments in microfluidic droplets

We used microfluidic dropmakers to encapsulate cells with LAMP components and lysis reagents into 500 pL droplets (Supplementary Figure S5). This dropmaker included two to three aqueous inlets depending on the experiment type. The device flow rates and inlet compositions are given in Supplementary Table S3. Supplementary Table S2 lists the final concentration of each logic gate for droplet logic experiments. We used standard LAMP primer concentrations, as described in ‘Bulk RT-LAMP Assays.’ In all experiments, we adjusted the cell concentration such that approximately one in every ten droplets contained a single cell. We collected droplets into a microcentrifuge tube and incubated at 65°C for 1 h prior to analysis. We then reinjected droplets onto a second microfluidic device with additional oil for spacing and measured their fluorescence in FAM/SYBR, HEX and Alexa Fluor 647 channels. We performed subsequent gating and analysis in Flowjo software. For single-target experiments, we omitted all LAMP primers as a negative control. For multiplexed experiments, we performed unamplified negative controls that were only incubated at 65°C for 5 min. We analyzed at least 10 000 cells for each replicate of each experiment, except for the droplet logic gate and *ESR1* experiments, where we analyzed over 1000 cells.

During the course of SNAPD development, we made several refinements to reduce false positive events. We switched from a 3-inlet droplet generator to a 2-inlet design. We also added RNase I_f_ (New England Biolabs) and DNase I (Thermo Fisher) to the cell suspension to destroy extracellular DNA and RNA. Likewise, we pre-treated the LAMP mastermix with DNase I for 30 min at room temperature, followed by a 30-min inactivation with Dithiothreitol (DTT) on ice, followed by primer addition. We used the 3-inlet, no-nuclease method for *ESR1*, droplet transducer, and droplet OR experiments, and the 2-inlet method with nuclease treatment for all other droplet experiments.

### Droplet fluorescence data processing

Our microfluidic measurements produce fluorescence time traces of the droplets passing the laser. We used a fixed threshold to distinguish droplets from the carrier oil, and the average fluorescence within each droplet was recorded at multiple wavelengths. The resulting data looks similar to flow cytometry data with many events across multiple fluorescence channels. For single-target experiments, we performed signal compensation to reduce crosstalk between the FAM/SYBR and HEX channels. We then gated out excessively large/small drops by examining the droplet size (duration of signal) and removing the top and bottom 8% of events (Supplementary Figure S7). Next, we selected the cell-containing droplet population by manually gating droplets displaying the calcein red–orange cell stain in the HEX channel. We then down-sampled 10 000 random events from the resulting droplet population to allow comparisons across samples. We defined a positive/negative LAMP amplification threshold as 2.8-fold higher than the median fluorescence of the empty droplet population. These same thresholds were applied to negative control conditions without LAMP primers. For the droplet logic experiment, we used a different optical configuration and different fluorophores, but used the same general compensation and gating strategy.

### RNA Flow-FISH

We performed assays in triplicate using the Primeflow RNA Assay Kit™ (Thermo Fisher) with Alexa Fluor 647 *ERBB2* target probes, following the recommended protocol. We stained MOLT-4 and SK-BR-3 cells with LIVE/DEAD™ Fixable Green Dead Cell Stain Kit, for 488 nm excitation (Invitrogen) prior to treatment. As a negative control, we also performed the entire protocol without the *ERBB2* target probes. We analyzed the samples on a BD Fortessa X-20 Flow Cytometer using APC and FITC channels, and performed subsequent gating and data analysis in FlowJo software (Supplementary Figure S7).

### Simulating SNAPD’s ability to detect rare cells

We used data from *ERBB2* limit of detection experiments (Figure 2D) to estimate SNAPD’s false positive rate (FPR) as 0.02%. Similarly, we used data from SK-BR-3 *ERBB2* amplification (Figure 2B) to estimate SNAPD’s true positive rate (TPR) as 97.1%. For each trace in Figure 2E, we fixed the number of cells analyzed, and simulated varying proportions of SK-BR-3 cells in a MOLT-4 background. In each simulation, we generated a binomial distribution to approximate the pure MOLT-4 population using the estimated FPR and the number of cells analyzed. We then calculated the expected number of observed positive cells to be equal to the number of MOLT-4 cells * FPR + the expected number of SK-BR-3 cells * TPR. We used this expected value to perform a binomial test against the pure MOLT-4 population and obtain a *P*-value for detecting positive cells.

### Enrichment of *ERBB2*± cells via microfluidic droplet sorting

We used droplet sorting to enrich *ERBB2+* cells from a cell mixture, and verified enrichment using RT-qPCR. We performed *ERBB2* SNAPD on a 9:1 mixture of MOLT-4 and SK-BR-3 cells stained with CellTrace™ Calcein Red-Orange AM (Invitrogen). We then reinjected these droplets onto a dielectric sorting device (Supplementary Figure S5) with flow rates of 100 μl/h for SNAPD droplets, 400 μl/h for reinjection oil and 1000 μl/h for bias oil. We measured the fluorescence of each drop in the FAM/SYBR and HEX channels. We sorted 350 positive droplets containing cells by applying a series of 250 800-V, 10-kHz DC pulses across the sorting junction. We performed sorting experiments in triplicate.

To verify enrichment, we extracted RNA from the sorted droplets and performed RT-qPCR on *GAPDH* and *KRT19*. We froze the sorted droplet samples at −20°C overnight to coalesce the emulsions and preserve RNA integrity. We then thawed samples, extracted the aqueous layer containing the RNA and pooled samples from the three sorting replicates to increase the RNA yield. For the initial (unsorted) samples, we used a 150 000 cells/ml suspension containing a 9:1 mixture of MOLT-4:SK-BR-3 cells, and serially diluted this mixture in 2-fold intervals to identify an RNA concentration that matched the sorted droplets. We subjected this cell mixture to bulk *ERBB2* RT-LAMP to match the experimental conditions of the sorted droplets. We then digested DNA in the sorted and initial (unsorted) samples by diluting 10-fold and adding 0.1 U/μl DNase I (Thermo Fisher) and 2 U/μl RNasin Plus RNase Inhibitor (Promega). We performed DNase digestion at 37°C for 1 h followed by a 95°C inactivation for 5 min. We added 1 μl of the resulting DNase digestions to 10 μl RT-qPCR reactions (Luna One-Step Universal RT-qPCR Kit, New England Biolabs) containing 0.8 U/μl RNasin Plus (Promega) and *GAPDH* or *KRT19* primers (primer sequences listed in Supplementary Table S1). For non-template controls, we added water instead of RNA. We performed thermocycling as recommended in the RT-qPCR kit protocol. We performed all RT-qPCR measurements in triplicate.

We calculated enrichment by evaluating how *KRT19* levels changed relative to the *GAPDH* reference gene. Since SK-BR-3 cells express the *KRT19* gene, a high *KRT19*/*GAPDH* ratio indicates SK-BR-3 enrichment relative to MOLT-4 cells. We derived an expression for enrichment based on a modified version of the standard 2^–ΔΔCT^ method. This expression accounts for varying PCR efficiencies between the target and reference gene (41). Enrichment was calculated as:

$$\begin{eqnarray*}E &=& \frac{{{X_{{\rm sorted}}}/{R_{{\rm sorted}}}}}{{{X_{{\rm inital}}}/{R_{{\rm initial}}}}}\nonumber\\ &=& {\left( {1 + {E_R}} \right)^{\left( {{C_{t,R,{\rm sorted}}} - \;{C_{t,R,{\rm initial}\;}}} \right)}} \cdot {\left( {1 + {E_X}} \right)^{\left( {{C_{t,X,{\rm initial}}} - \;{C_{t,X,{\rm sorted}}}} \right)}}\end{eqnarray*}$$

where *X*_sorted_ and *R*_sorted_ correspond to target (*KRT19*) and reference (*GAPDH*) RNA levels in the sorted sample, respectively; and *X*_inital_ and *R*_inital_ correspond to the initial sample. *C_t,_*_R,sorted_ is the number of PCR cycles for the reference gene to reach a defined threshold in the sorted sample, *C_t,_*_R,initial_ corresponds to *GAPDH* in the initial sample, *C_t,X_*_,sorted_ corresponds to *KRT19* in the sorted sample and *C_t,X_*_,initial_ corresponds to *KRT19* in the initial sample. *E_R_* and *E_X_* are the PCR efficiencies for the reference and target, respectively; and were estimated to be *E_GAPDH_* = 0.36 and *E_KRT19_* = 0.17 by running RT-qPCR assays at varying cell lysate concentrations.

### Optical configurations used in droplet experiments

We used two custom-built optical setups for droplet experiments, as shown in Supplementary Figure S8a and b. For the orthogonal droplet transducer experiment, we used the four-color setup depicted in Supplementary Figure S8b. For all other droplet experiments, we used the three-color setup shown in Supplementary Figure S8a. Individual lasers (Thor Labs, Supplementary Figure S8a) or a combination unit with four lasers (Changchun New Industries Optoelectrics, Supplementary Figure S8b) were redirected and filtered via a set of dichroic mirrors and bandpass filters (Semrock) to illuminate the microfluidic device on a standard brightfield microscope (Lukas Microscopes Services Inc.) at 10×–40× magnification. We monitored droplet processing using a high-speed camera (Vision Research). During droplet analysis, fluorescence was passed through a set of dichroic mirrors and bandpass filters (Semrock) and detected by a set of photomultiplier tubes (Thor Labs) and analyzed in LABVIEW software. For sorting experiments, we used a Trek PZD700A high-voltage amplifier to redirect droplets. Wavelengths and specifications for each optical setups are shown in Supplementary Figure S8a and b.

### Statistical testing

All *P*-values reported in this work were performed using a two-sided *t*-test with *n* = 3, assuming homoscedasticity.

## RESULTS

### A microfluidic platform for transcriptional profiling of single cells

**Figure 1.**
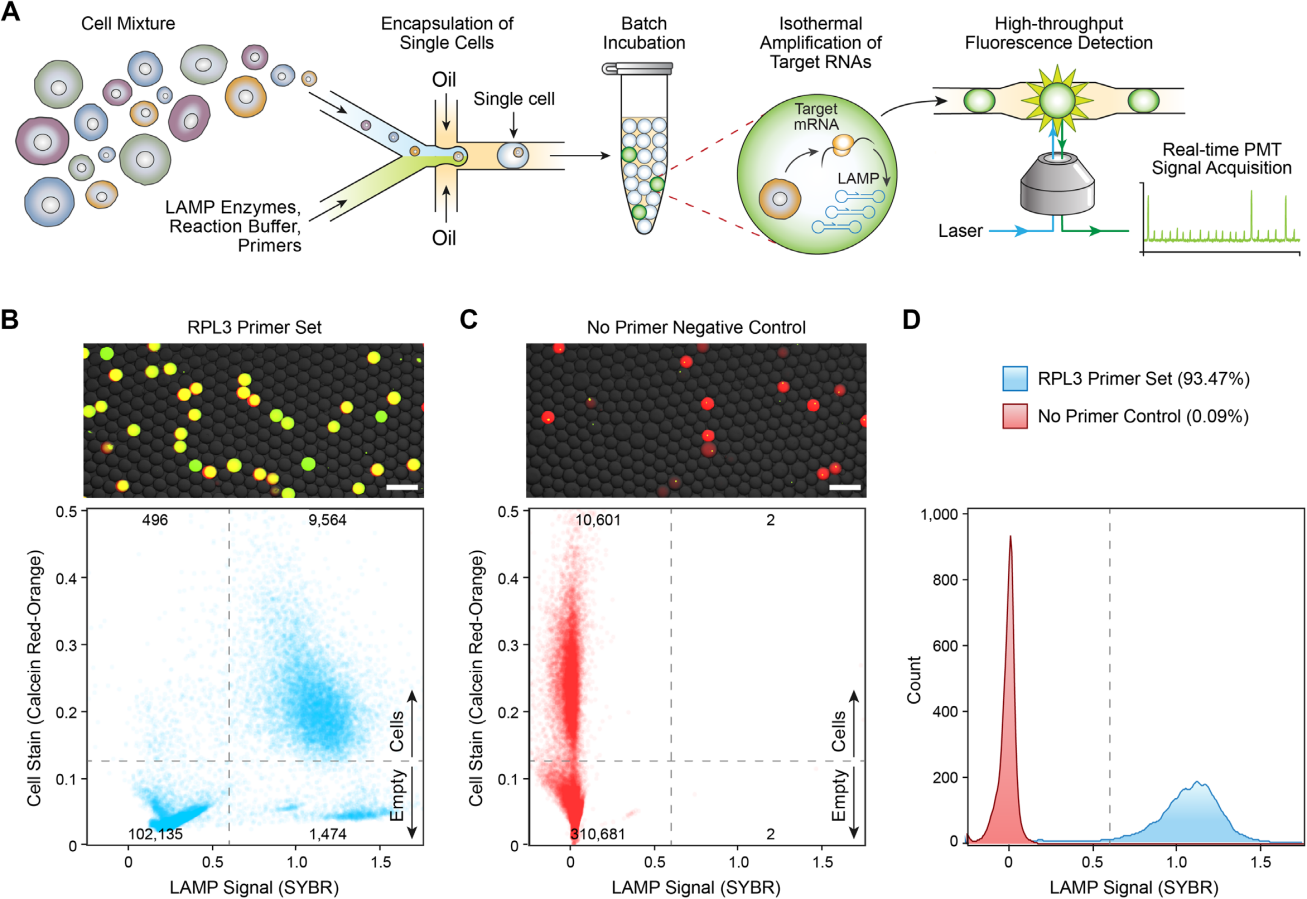
A microfluidic platform for high-throughput single-cell RNA profiling. (**A**) Schematic of the SNAPD workflow. Single cells are encapsulated into microdroplets with assay reagents, collected and incubated offline, and the fluorescence of each droplet is subsequently measured to indicate amplification of target RNAs. SNAPD can analyze 300 droplets per second. (**B**) Microscopy image and scatterplot of SNAPD droplet fluorescence. We assayed MOLT-4 cells for expression of the housekeeping transcript *RPL3*. Red fluorescence indicates the presence of a cell, while the green dye indicates target amplification. Droplets that appear yellow in the microscopy image contain cells and also displayed *RPL3* amplification. Scale bars indicate a length of 200 μm. The numbers on the scatter plot indicate the number of droplets in each fluorescence quadrant. (**C**) Microscopy images and scatterplot of a SNAPD negative control with *RPL3* primers omitted. Scale bars indicate a length of 200 μm. (**D**) Histograms of the cell-containing droplets SYBR fluorescence. On average, reactions containing the *RPL3* primer set (blue) displayed 93.5% amplification, while the no primer negative control (red) only amplified at 0.1%. These results demonstrate that SNAPD can reliably detect specific mRNA targets in single cells with high specificity.

We developed SNAPD to perform RT-LAMP-based detection of specific mRNA transcripts from single cells (Figure 1A). The SNAPD workflow is simple, rapid and requires minimal hands-on processing. Live cells are stained with a live/dead indicator dye and loaded directly into a microfluidic device. Single cells are encapsulated into droplets containing RT-LAMP and lysis reagents, along with target-specific primer sets. Droplets are then collected and incubated at a constant temperature, allowing the RT-LAMP reaction to proceed. Finally, the droplets are reinjected onto a second microfluidic device, where their fluorescence is analyzed in high-throughput to detect RT-LAMP-based amplification of the target markers.

We first benchmarked our SNAPD platform by measuring single-cell expression of the 60S Ribosomal Protein L3 (*RPL3*) housekeeping transcript. *RPL3* is known to be uniformly expressed across single bone marrow progenitor cells (42), and thus, we used the leukocytic leukemia line MOLT-4 as a positive control. We performed SNAPD on MOLT-4 cells with an *RPL3* primer set, and also performed a no-primer negative control (Figure 1B and C). Microscopy showed that cells (red) were loaded into ∼10% of droplets, as expected, and nearly all cell-containing droplets displayed *RPL3* amplification as visualized by SYBR green. In contrast, the no-primer negative control experiment displayed no SYBR green signal, indicating that RT-LAMP-based mRNA amplification was responsible for the SYBR green signal in the cell-containing droplets.

We reinjected the SNAPD droplets onto a high-throughput fluorescence detection device, and measured the fluorescence of 10,000 cells. A majority of drops displayed a strong correspondence between the cell stain and RT-LAMP amplification. There were a small fraction of drops that displayed RT-LAMP amplification in the absence of cells, presumably due to free transcripts or dead cells that weren’t stained. There were also a small fraction of drops that displayed stained cells with no RT-LAMP amplification. We gated the cell-containing droplets, and found that 93.5% of MOLT-4 cells amplified with the *RPL3* primer set, as compared to 0.1% for the no-primer negative control (Figure 1D). These results demonstrate that SNAPD can reliably detect specific mRNA targets in single cells. We were able to perform droplet generation and analysis at a rate of 300 Hz, allowing SNAPD to transcriptionally characterize over 100,000 cells per hour.

### Quantifying single-cell gene expression in heterogeneous cell populations

**Figure 2.**
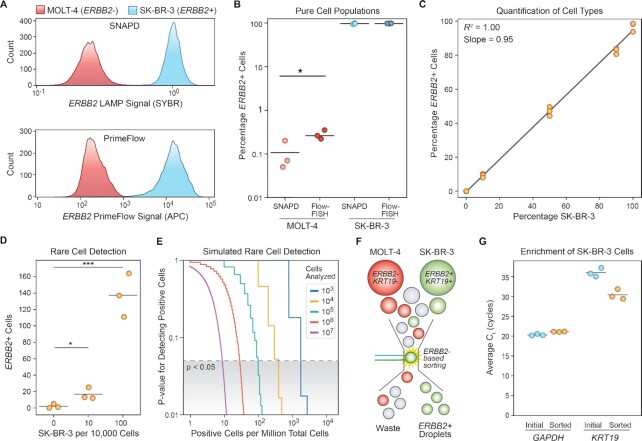
Quantification and enrichment of specific cell types. (**A**) SNAPD and PrimeFlow™ histograms comparing *ERBB2* (HER2) expression in MOLT-4 and SK-BR-3 cells. The overall fluorescence distributions are nearly identical, indicating SNAPD is comparable with well-established and commercially available single-cell analysis methods. (**B**) The percentage of *ERBB2*+ cells as measured by SNAPD and PrimeFlow™. Experiments were performed in triplicate. There was no significant difference between SNAPD and PrimeFlow™ when analyzing SK-BR-3 cells, while SNAPD displayed a lower FPR when analyzing MOLT-4 cells (*P* < 0.05). (**C**) SNAPD quantification of *ERBB2+* cells in mixtures of MOLT-4 and SK-BR-3 cells at varying proportions. Each measurement was performed in triplicate. These results indicate that SNAPD can quantify specific cell types across a broad range of proportions with high accuracy and precision. (**D**) SNAPD can detect rare *ERBB2+* cells in mixtures containing low proportions of SK-BR-3 cells spiked into MOLT-4 cells. All experiments were performed in triplicate. A mixture containing 0.1% SK-BR-3 cells was distinguishable from a sample of pure MOLT-4 cells (*P* < 0.05), indicating a limit of detection below 1 in 1000 cells. (**E**) Simulation results exploring how the total number of cells analyzed affects SNAPD’s ability to detect rare cells. Analyzing 100 000 cells should be sufficient to detect positive cells at a prevalence of 1 in 10 000. (**F**) SNAPD can enrich specific cell types from mixtures using microfluidic sorting. MOLT-4 and SK-BR-3 cells were mixed at a 9:1 ratio, analyzed for *ERBB2* expression using SNAPD and droplets displaying *ERBB2* amplification were isolated using a microfluidic droplet sorting device. (**G**) SNAPD-based cell enrichment of SK-BR-3 cells, validated via RT-qPCR on the SK-BR-3-specific *KRT19* marker. RT-qPCR measurements were performed in triplicate. * *P* < 0.05, ** *P* < 0.01, *** *P* < 0.001.

Single cell analysis methods can be used to distinguish different cell types and characterize heterogeneous cell populations. We used SNAPD to evaluate the expression of the HER2 (*ERBB2*) breast cancer marker in two cell lines. The SK-BR-3 breast cancer line overexpresses this gene, while the MOLT-4 leukemia line has no detectable expression (39). We performed SNAPD on pure cell lines and found 97.1% of the SK-BR-3 cells displayed *ERBB2* expression, as opposed to 0.1% of the MOLT-4 cells (Figure 2A and B). We also tested whether SNAPD could distinguish different cell types from the same tissue of origin by evaluating estrogen receptor (*ESR1*) expression in SK-BR-3 and MCF7 breast cancer lines. We found *ESR1* was expressed in 1.9% of SK-BR-3 and 67.9% of MCF7 cells (Supplementary Figure S2). *ESR1* is known to be heterogeneously expressed across single cells (43).

To further validate these results, we benchmarked SNAPD against Thermo Fisher's PrimeFlow™ RNA assay kit, a well-established and commercially available Flow-FISH method to measure gene expression in single cells. PrimeFlow™ detects RNA by fixing cells, hybridizing fluorescent probes, washing away excess probes and analyzing the target RNA within each cell by flow cytometery. SNAPD and PrimeFlow™ displayed highly similar *ERBB2* gene expression profiles across the two cell lines (Figure 2A and B). There was no statistically significant difference between the SK-BR-3 (*ERBB2+*) detection rates of the two methods, although SNAPD did show a slightly lower MOLT-4 (*ERBB2-*) cell line amplification rate with statistical significance (*P* < 0.05). This implies that SNAPD can reliably identify *ERBB2* positive cells with a lower FPR than PrimeFlow™. We observed a small number of false positives in negative controls lacking *ERBB2* primers, which we believe are caused by cellular autofluorescence or events where the laser directly strikes a cell's nucleus (Supplementary Figure S3).

After verifying that SNAPD could distinguish different cell types, we evaluated its ability to quantify specific cell types within a heterogeneous cell population. To do this, we combined SK-BR-3 and MOLT-4 in varying proportions, and used SNAPD to analyze the *ERBB2* marker in these defined cell mixtures. We found that SNAPD could reliably quantify the percentage of SK-BR-3 cells with a near-perfect linear fit (*R*^2^ = 1.00) and a slope of 0.95 (Figure 2C). SNAPD provides a highly quantitative readout of a population's cell types based on gene expression.

Single-cell methods can provide valuable information about rare cell types within a population. We tested SNAPD’s ability to detect rare cells by spiking a small known quantity of SK-BR-3 cells into MOLT-4 suspensions, and counting the resulting number of *ERBB2*+ cells within the sample. SNAPD could reliably count SK-BR-3 cells in mixtures containing as few as 10 SK-BR-3 cells per 10,000 total cells analyzed, and could distinguish these mixtures from a negative control containing 100% MOLT-4 cells (Figure 2D). SK-BR-3 cells at a prevalence of 1 in 10,000 total cells analyzed could not be distinguished from the negative control (Supplementary Figure S4). In this case, the total number of cells analyzed (10,000) limits SNAPD’s ability to detect rare cells. We performed statistical tests based on the observed FPR and TPR to explore how the total number of cells analyzed affects rare cell detection (Figure 2E). In each test, we calculated the expected number of observed positive cells from a cell mixture and used a binomial test to compare it to the expected binomial distribution for the pure MOLT-4 population. From these tests, we estimate our *ERBB2* SNAPD assay should be able to detect positive cells at a prevalence of 1 in 10,000 by screening only 100,000 cells total. Based on these results, SNAPD is capable of enumerating specific cell types from mixtures based on their RNA content, even when these target cells are rare.

The SNAPD single-cell assay can be combined with high-throughput droplet sorting to isolate specific cell types based on their RNA biomarker status. With a droplet microfluidic sorting device adapted from a previous design (Supplementary Figure S5) (36), we used the *ERBB2* marker to enrich SK-BR-3 cells from an initial population containing 90% MOLT-4 and 10% SK-BR-3 cells (Figure 2F). We then performed RT-qPCR on the initial and sorted pools to verify enrichment of SK-BR-3 cells. We used *GAPDH* as a reference gene, and estimated the samples’ proportion of SK-BR-3 cells by measuring the relative expression of the SK-BR-3-specific marker *KRT19*. We found the *GAPDH* levels were similar between samples, while the sorted sample contained higher *KRT19* levels, indicating a higher proportion of SK-BR-3 cells (Figure 2G). We observed poor amplification efficiency in RT-qPCR reactions, and therefore we could not accurately quantify enrichment values using standard approaches. We estimated enrichment by taking each target's observed RT-qPCR efficiency into account following established RT-qPCR calculations (41) and by assuming *GAPDH* expression in SK-BR-3 cells is greater than or equal to its expression in MOLT-4 cells (39). With these assumptions, we estimate that microfluidic sorting achieved at least a 3.2-fold enrichment of SK-BR-3 cells over MOLT-4 cells. These results demonstrate that SNAPD can be combined with microfluidic droplet sorting to enrich target cell types from a heterogeneous population.

### Multiplex SNAPD to profile multiple RNA targets

Analyzing multiple RNA targets in single cells would allow SNAPD to identify more complex cellular phenotypes and classify cells with greater selectivity and sensitivity. Multiplex SNAPD requires simultaneous RT-LAMP-based amplification of multiple RNA targets, in addition to orthogonal fluorescent readouts of each amplified target. Coupling RT-LAMP amplification to a specific fluorescence signal is challenging due to the unpredictable and heterogeneous mix of concatemers generated by LAMP (25).

**Figure 3.**
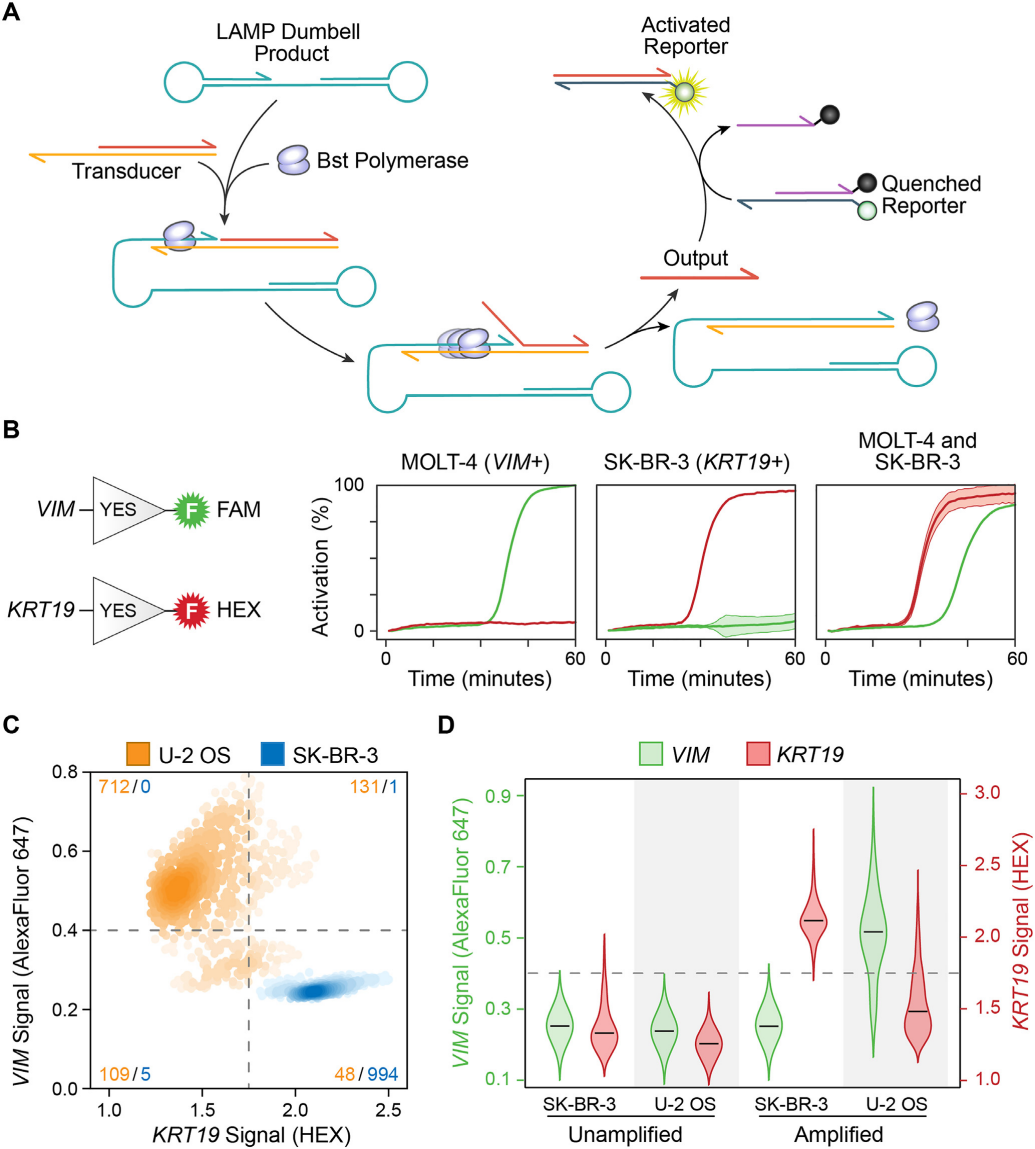
Multiplexed SNAPD profiles multiple mRNA targets in single cells. (**A**) Molecular transducer that converts the LAMP dumbbell product to a fluorescent readout in a sequence-specific manner. The *Bst* polymerase that is present in the RT-LAMP reaction drives the displacement of the output strand; this output strand can then activate a fluorogenic reporter through toehold-mediated strand displacement. (**B**) Sequence-specific molecular transducers can operate orthogonally to link amplification of specific RNA targets to unique fluorescent outputs. We designed transducers that produce FAM fluorescence in response to *VIM* amplification and HEX fluorescence in response to *KRT19*. We added LAMP primer sets targeting *VIM* and *KRT19* and the two molecular transducers to lysates from either MOLT-4 or SK-BR-3 cells. The fluorescence of each reaction was monitored over time. The MOLT-4 lysate displayed specific activation of the VIM channel, while the SK-BR-3 lysate activated the KRT19 channel. A mixture of the two lysates activated both channels. We performed each measurement in triplicate. (**C**) Simultaneous profiling of *VIM* and *KRT19* expression in single cells using SNAPD. We combined the orthogonal *VIM/KRT19* transducers and SNAPD to analyze SK-BR-3 cells (blue) and the mesenchymal cell line U-2 OS (orange). Numbers of each cell type within each quadrant are indicated. 99.4% of SK-BR-3 cells occupy the *VIM-/KRT19+* quadrant, while 71.2% of U-2 OS cells fall into the *VIM+/KRT19-* quadrant. (**D**) Violin plots showing the marginal distributions of *VIM* and *KRT19* signal in a multiplexed SNAPD experiment. SK-BR-3 cells display low *VIM* and high *KRT19* expression, while U-2 OS cells display high *VIM* and low *KRT19*. Also shown are the droplet fluorescence distributions before amplification to demonstrate the observed signals are a result of RT-LAMP.

We designed a polymerase-driven DNA strand displacement scheme to detect RT-LAMP products in a sequence-specific manner (Figure 3A). The goal was to build a molecular transducer that would convert a LAMP dumbbell product into a short DNA oligo that could then activate a fluorogenic reporter, or be fed into additional downstream strand displacement reactions. The molecular transducer consists of a ‘gate’ strand hybridized with an ‘output’ strand to produce a DNA duplex with a 3′ overhanging ‘toehold’. The LAMP dumbbell can hybridize to the transducer's toehold, and its 3′ end acts as a primer for the polymerase to replicate the gate strand. The polymerase displaces the output strand during extension, releasing it. The free output strand can then activate a quenched fluorogenic reporter by displacing the quencher strand from the fluorophore strand. By designing a unique transducer for each LAMP product and a unique reporter for each transducer, we can link each RNA target's amplification to a unique fluorophore. This detection strategy allows flexible multiplexing with minimal crosstalk.

We tested our LAMP detection scheme and its ability to be multiplexed by designing two orthogonal amplification/transducer/reporter systems. The first produces a FAM signal in response to the mesenchymal marker vimentin (*VIM*), and the other produces a HEX signal in response to the epithelial marker cytokeratin 19 (*KRT19*). We added these orthogonal transducers to lysate from MOLT-4 (*VIM*+/*KRT19*-) and SK-BR-3 (*VIM*-/*KRT19*+) cells. As designed, the MOLT-4 lysate produced a FAM signal only, the SK-BR-3 produced a HEX signal only and a 1:1 mixture of the lysates activated both channels (Figure 3B). These results demonstrate that LAMP can amplify multiple targets in highly concentrated cell lysate, and that our transducer designs operate orthogonally to activate two separate fluorophores without signal crosstalk.

We combined our orthogonal LAMP transducers and SNAPD to evaluate the expression of *KRT19* and *VIM* in single cells. These two markers indicate a cell's position along the epithelial-mesenchymal axis, and thus provide valuable information about cellular differentiation and cancer invasiveness (44). We analyzed the epithelial-derived SK-BR-3 (*VIM*-/*KRT19*+) cells and found that 99.4% fell into the *VIM*-/*KRT19*+ quadrant (Figure 3C and D). We also performed an unamplified negative control to identify the signal background. Next, we analyzed the mesenchymal cell line U-2 OS (*VIM*+/*KRT19*-) and found that 71.2% fell into the *VIM*+/*KRT19*- quadrant. These results demonstrate that SNAPD can be multiplexed and used to profile multi-gene phenotypes in single cells.

### Analysis of complex phenotypes with molecular logic

Cellular phenotypes often depend on expression profiles across a multitude of genes. Our transducer-based multiplexing strategy is highly scalable; however, crowding within the optical spectrum restricts the number of orthogonal fluorescent reporters. To overcome these limitations, we devised a molecular logic scheme to integrate signals from multiple LAMP reactions and return a single fluorescent output based on a multi-level logical computation.

**Figure 4.**
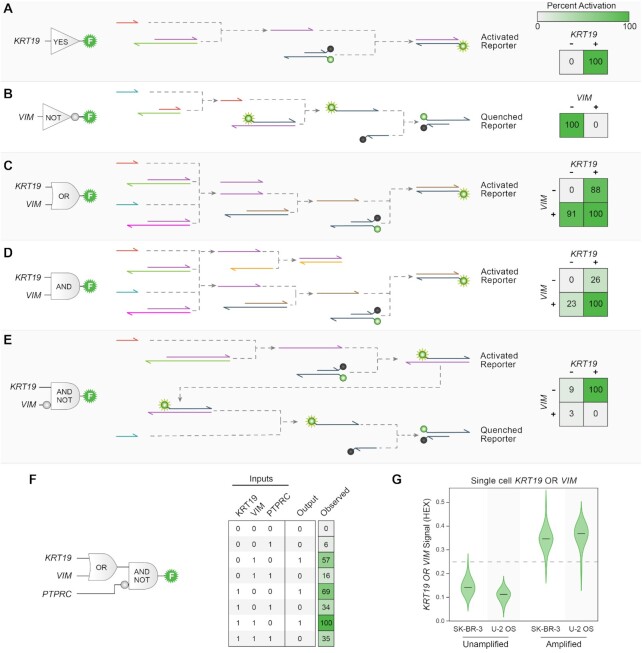
Molecular logic gates integrate multiple LAMP signals. (**A**–**E**) Molecular logic gate designs for YES, NOT, OR, AND and AND-NOT operations. The designs leverage *Bst* polymerase's strand displacement activity to drive the reaction cascade. We tested the designs on various combinations of purified RNA targets and all designs displayed the correct truth tables with a high signal-to-background ratio. Colors and values within each truth table square indicate the normalized percent activation from the fluorogenic probe. (**F**) Multi-level logic circuit that integrates RT-LAMP signals from *KRT19*, *PTPRC* and *VIM* targets. The circuit displayed the intended logical outputs based on all 2^3^ combinations of inputs. (**G**) Integrating information across multiple markers in single cells. We applied a molecular OR gate to combine *KRT19* and *VIM* signals from single SK-BR-3 or U-2 OS cells in a SNAPD experiment. As expected, both cells activated the molecular OR gate, while an unamplified negative control displayed low fluorescence levels.

We used the *Bst* polymerase-driven strand displacement mechanism to design molecular logic gates comprising YES, NOT, OR, AND and AND-NOT operations (Figure 4A–E and Supplementary Figure S6). These designs build off of the LAMP transducer mechanism described in the previous section (Figure 3A). The YES gate is simply a transducer and converts a LAMP product to a fluorescent output. The NOT gate inverts a LAMP signal by passing the output of a transducer to displace an unquenched fluorophore, which can then hybridize to a strand containing a quencher. The OR gate is composed of two transducers that accept different input sequences, but produce identical output strands. The presence of either LAMP signal will produce the output and generate a fluorescent signal. The AND gate similarly incorporates two transducers, but also contains a ‘threshold strand’ which sequesters the transducers’ output. When only one of the transducers is activated, the threshold strand sequesters all of the output; however, activation of both transducers produces a stoichiometric excess of the output strand over the threshold strand, leaving a sufficient amount of output strand to activate the fluorogenic reporter. Thus, a fluorescent signal is only produced when both LAMP signals are present. The AND-NOT gate is composed of a transducer that generates a fluorescent reporter in response to the first LAMP input, and is then followed by a not gate that inverts this signal in response to the second LAMP input. This gate will produce a fluorescent signal if the first input is on and the second input is off. This set of molecular logic gates is functionally complete, so in principle individual gates can be combined to produce any conceivable logical operation.

We constructed molecular logic gates to detect the presence of *VIM* and *KRT19* signals, and tested their ability to produce the expected truth tables. We added the molecular logic gates to RT-LAMP reactions containing various combinations of purified RNA targets, and found the gates produced the desired logic with a high signal-to-background ratio. Across all gates, the average ON signal was 1.3-fold higher than the average OFF signal. The AND gate showed the lowest signal-to-background ratio with a 1.1-fold difference between the average ON state and the average OFF state.

We next built a multilevel logic cascade to integrate three LAMP inputs and activate a single fluorescent reporter (Figure 4F and Supplementary Figure S6). We designed this circuit to implement *(KRT19* OR *VIM*) AND-NOT *PTPRC* (CD45), a logical operation which could be used to distinguish epithelial or mesenchymal circulating tumor cells (CTCs) from leukocytes in blood samples (45). We found that this circuit correctly identified each combination of RNA inputs with a signal-to-background ratio of 1.2. This demonstrates that our molecular logic gates can be stacked for profiling higher-dimensional cell phenotypes.

We combined the molecular OR gate and SNAPD to evaluate whether single cells express *KRT19* or *VIM* (Figure 4G). As expected, both SK-BR-3 cells (*KRT19*+/*VIM*-) and U-2 OS cells (*KRT19*-/*VIM*+) produced a fluorescent output. This shows that our LAMP based logic gates can analyze single cells in droplet reactions. Together, these logic gates provide a powerful means to enhance SNAPD and enable multiple transcripts to be profiled with a single fluorescence output.

## DISCUSSION

Multicellular organisms are composed of hundreds of distinct cell types that interact to drive physiological, developmental, and disease processes. Single-cell analysis methods provide a valuable means to study specific cell types within heterogeneous cell populations. Single-cell approaches have been applied to a myriad of biological topics: dissecting heterogeneity in solid tumors (46), elucidating drivers of cell fate during embryogenesis (47) and reconstructing transcriptional regulatory networks (48), among others. In this work, we developed SNAPD to analyze RNA biomarker expression in tens of thousands of single mammalian cells. SNAPD provides a simple, low cost, rapid and scalable approach to detect subtypes within heterogeneous cell populations.

Existing single-cell transcriptomic methods include single-cell RNA Sequencing (scRNA-seq), single-cell qPCR (sc-qPCR), *in situ* hybridization methods such as Flow-FISH and PCR-Activated Cell Sorting (PACS) in droplets. While methods such as scRNA-seq and sc-qPCR can analyze thousands of different transcripts, they suffer from expensive workflows and long turnaround times. On the other end of the spectrum, Flow-FISH and PACS profile smaller numbers of transcripts across millions of cells (19). However, Flow-FISH and PACS both suffer from expensive and complex workflows as well. SNAPD occupies a unique position among single-cell analysis methods by providing an inexpensive, rapid and simple single-cell enumeration process. SNAPD achieves a high throughput with over 100,000 cells per hour, comparable to Flow-FISH and PACS. SNAPD’s analysis throughput could be reasonably scaled tenfold by optimizing the droplet size and device flow rates (36,49). Furthermore, when combined with molecular logic, SNAPD can detect multiple transcripts and integrate these signals to produce a single fluorescence output. In principle, this molecular logic strategy could be scaled to process 10s of transcriptional inputs. Finally, SNAPD’s experimental workflow is highly streamlined, requires minimal hands-on processing, and provides results within a few hours.

We evaluated SNAPD’s ability to detect specific cell types with high sensitivity and specificity by analyzing defined mixtures of *ERBB2*-positive and -negative cells. We found SNAPD could reliably quantify the proportion of positive cells across a range spanning from 0.1% to 100%. Furthermore, we compared SNAPD to Thermo Fisher's well-established Flow-FISH (PrimeFlow™) kit and found the two methods detected the same number of *ERBB2*-positive cells. However, SNAPD displayed nearly a 3-fold lower false positive rate (FPR) for *ERBB2*-negative cells, suggesting that it has substantially higher specificity than Flow-FISH. This low FPR allowed us to detect rare cells from mixed populations with high sensitivity and specificity. We successfully detected *ERBB2*-positive cells at 0.1% prevalence in a mixture, suggesting SNAPD’s limit of detection is less than 1 target cell per 1,000 total cells. Based on our results, we predict that *ERBB2*-positive cells could be reliably detected at a prevalence of 0.01% if the number of analyzed cells were increased from 10,000 to 100,000 (Figure 2E). This level of sensitivity would be sufficient for numerous rare cell detection applications, including isolating mesenchymal stem cells from bone marrow (50), pericytes from adipose tissue (51) and breast cancer progenitors from pleural effusions (51). These are therefore feasible applications for our SNAPD platform with scaled up throughput.

In this work, we developed a novel multiplex RT-LAMP detection scheme to analyze multiple RNA targets simultaneously. Previous efforts to perform sequence-specific detection of LAMP products have relied on a branch migration-based strand displacement mechanism targeting the loop region of the LAMP dumbbell structure (52,53). Our LAMP detection scheme also uses a strand displacement mechanism, but takes advantage of the LAMP reaction's *Bst* polymerase to drive strand displacement using the 3′ end of the LAMP product as a primer. The displaced strand can then displace a DNA duplex containing a fluorophore:quencher pair, thereby producing a fluorescent signal. Polymerase-driven strand displacement should display faster kinetics than branch migration because it does not rely on a random-walk strand exchange process (54,55). In addition, the rate of polymerase-driven strand displacement depends only linearly on the length of the transducer's duplex region, allowing longer duplex domains that result in less background leakage. Our polymerase-driven strand displacement scheme enables fast, robust, and orthogonal detection of specific LAMP products, even in complex mixtures. This capability was essential for our SNAPD platform to analyze the expression of multiple transcripts from single cells, but could also find more general applications in point-of-care nucleic acid detection.

We designed and implemented a novel set of DNA logic gates to integrate multiple LAMP signals into a single fluorescent output. Our designs leverage *Bst* polymerase activity to drive strand displacement, resulting in simple and robust DNA logic gates with fast kinetics. A similar DNA logic approach was recently described that used *Bst* polymerase to drive chemical reaction networks consisting of single-stranded oligos and primers (56). Individual DNA logic gates can be combined into multi-input, multi-layer circuits that perform complex logical operations on nucleic acid inputs (56–59). This would allow us to design logic circuits to identify very specific cell types based on the expression of multiple genes. DNA logic gates can also be used to build digital encoders that convert *2^n^* binary inputs to *n* distinct outputs (60). We could apply digital encoders to profile gene expression states of 16 different transcripts using only four fluorescence channels. This would allow enumeration of large numbers of distinct cell phenotypes in heterogeneous populations, while maintaining a simple optical detection setup. The modularity, flexibility, and robustness of our polymerase-driven DNA logic gates provides avenues for numerous SNAPD applications.

Our SNAPD single-cell analysis platform has several limitations that could be addressed in future iterations. First, we found RT-LAMP primer design to often require screening multiple designs to identify a primer set with fast amplification and low background. Some of this variability is likely caused by mRNA secondary structure or the specific splice isoforms present in each cell type. To overcome this, we found it was useful to include two redundant RT-LAMP primer sets targeting each transcript. This strategy increased the overall RT-LAMP signal and resulted in a lower false negative rate in SNAPD. To ensure successful amplification, we also targeted exonic regions shared between highly expressed splice variants. However, a systematic primer design framework that considers mRNA secondary structure across splice isoforms may improve the reliability of our method.

Another potential limitation involves measuring poorly expressed mRNA targets, an issue which is common to single-cell RNA sequencing assays (61). Some markers may only have 1–10 transcripts per cell and result in low femtomolar concentrations when diluted into a ∼500 pL droplet. RT-LAMP assays have achieved sub-femtomolar and even attomolar detection of nucleic acids (62,63). However, it is unclear whether this high level of sensitivity will translate to our droplet assay, which operates in dense cell lysate and has a high surface-to-volume ratio due to the microemulsion environment. Many of the transcriptional markers tested in this work are considered highly expressed, with normalized expression (NX) values ranging from 49.5 to 284.6 (39). The lowest expressed marker tested was *ESR1*, with an NX value of 24.3 in MCF7 cells. This is only 5% of the highest expressed gene in the MCF7 cell line (39). Future work should explore SNAPD’s ability to measure lowly expressed transcripts, and compare it to the high dropout rates observed in single-cell RNA-seq.

Many applications require isolating a specific cell type from a population; this enriched sample can then be analyzed using other methods such as mass-spectrometry or RNA-seq. In this work, we integrated our single-cell analysis technique with high-throughput droplet sorting to isolate specific cell types based on their RNA biomarker expression, achieving an enrichment of ∼3.2-fold. These results demonstrate that SNAPD can be combined with microfluidic droplet sorting, providing some added flexibility to our method. However, the observed enrichment ratio was suboptimal and the resulting RNA was scarce and challenging to analyze using standard methods. We suspect this may result from RNA degradation during the LAMP process due to slightly alkaline buffer conditions, elevated temperatures, and potential RNase activity. The enrichment ratio and RNA scarcity we observed could also result from errors in the sorting process. If flow rates are not perfectly equilibrated, off-target droplets can be diverted into the sorted droplet channel. We would expect empty droplets to dilute the captured RNA while droplets containing negative cells would lower the enrichment ratio. Thus, our technology is not currently compatible with downstream RNA-dependent applications. However, this could be feasible upon further optimization of RNA preservation and sorting processes.

A final limitation involves the detection of ultra-rare cells such as circulating tumor cells (CTCs). CTCs are typically found in blood samples at frequencies on the order of one CTC per 10 million nucleated blood cells (64,65). We estimate that SNAPD is currently appropriate for enumerating rare cells at frequencies of one target cell per 1,000 total cells. To detect CTCs, we would need to reduce SNAPD’s FPR by approximately three orders of magnitude. We believe many false positive events arise when a cell nucleus passes through the laser, causing a large spike in the SYBR signal due to the genomic DNA. This could be addressed by using transducers instead of intercalating dyes, processing the time traces to filter large spikes, or examining the emission spectra in multiple channels to correct for cellular auto fluorescence (66). Another source of false positive events came from amplification of extracellular RNA and/or DNA, which has been observed previously in droplet PCR (18,19). We reduced the impact of these rogue nucleic acids by adding RNase I and DNase I to the input cell suspension, and then inactivated the nucleases with reducing agents during cell encapsulation into droplets. We also pre-treated the RT-LAMP mastermix solution with DNase I to eliminate product contamination, and quenched with reducing agents prior to the assay. We found this approach reduced the FPR as much as 100-fold without reducing the true positive rate (TPR). Additional nucleases and other additives may decrease the FPR even further. Despite these various sources of false positive events, SNAPD remains a highly selective assay with an overall FPR that is significantly lower than established Flow-FISH methods.

Increasing SNAPD’s cell analysis throughput would improve rare cell detection and provide a more comprehensive view of heterogeneity within cell populations. The throughput of our system is currently limited by the microfluidic fluorimeter device, which processes droplets at a rate of ∼300 Hz. Attempts to operate this device at faster flow rates resulted in droplet shredding due to the large size of the drops relative to the microfluidic channels. The system's throughput could be increased by either redesigning the microfluidic device to better accommodate large droplets or by decreasing the droplet volume. We initially chose a relatively large 500 pL droplet size to mitigate LAMP reaction inhibition by concentrated cell lysate. However, lysate titration experiments (Supplementary Figure S1) suggest that we could decrease droplet sizes to at least 100 pL, which would increase the screening throughput 5-fold. Decreasing the droplet size has the additional benefits of reducing reagent cost and analysis time, thereby further enabling larger samples and more accurate rare cell detection.

Experimental methods that capture properties of individual cells across large heterogeneous populations are essential for understanding the intricate and complex behaviors of biological systems. Methods to enumerate cells based on RNA biomarkers are currently hampered by long workflows and expensive reagents. In this work, we developed the SNAPD platform to address the limitations of existing single-cell analysis methods. SNAPD is simple, rapid, low cost and scalable, making it a powerful approach to characterize RNA targets at the single-cell level. With these advantages, SNAPD could help lower the barrier to entry for single-cell methods and allow widespread adoption among life science researchers.

## DATA AVAILABILITY

Microfluidic designs are available at https://github.com/RomeroLab/microfluidic-designs. The data that support the findings of this study are available from the corresponding author upon reasonable request.

## Supplementary Material

gkab577_Supplemental_File
