# Supplementary material for: Single-cell nucleic acid profiling in droplets (SNAPD) enables high-throughput analysis of heterogeneous cell populations
Source: Nucleic Acids Res. 2021 Jul 7;49(18):e103. doi: 10.1093/nar/gkab577 (PMC8501953; doi:10.1093/nar/gkab577)
Supplement: gkab577_Supplemental_File [file gkab577_supplemental_file.docx]

**SUPPLEMENTARY INFORMATION**

**
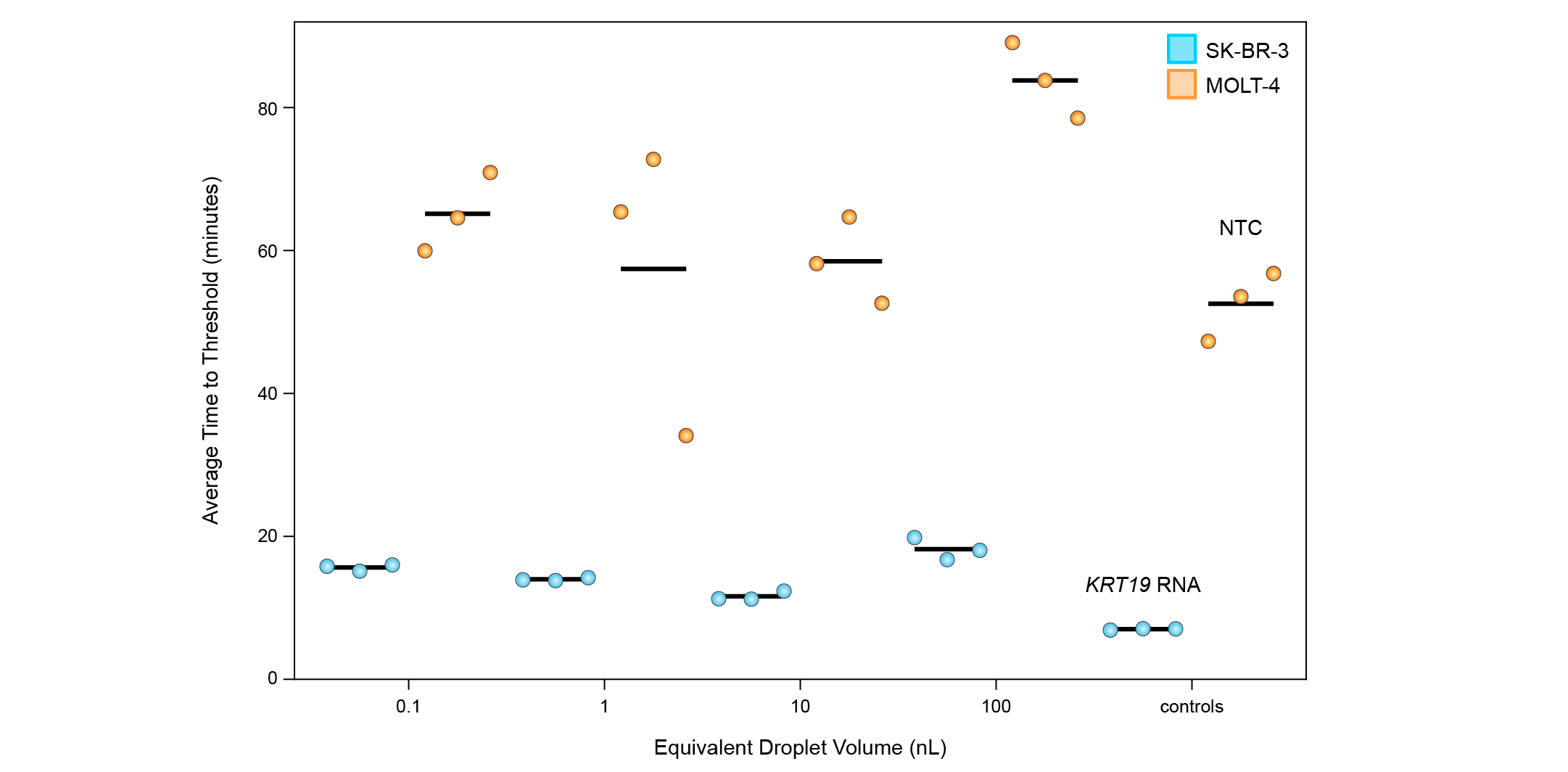
**

**Supplementary Figure 1.** *KRT19* LAMP experiments in various concentrations of target (SK-BR-3) and off-target (MOLT-4) cell lysate. Experiments were performed in triplicate. *In vitro* transcribed *KRT19* mRNA was used as a positive control. LAMP is highly tolerant of cell lysate, functioning in conditions equivalent to those of a 100pL droplet. However, a concentration equivalent to 1nL droplets yields faster detection.

**
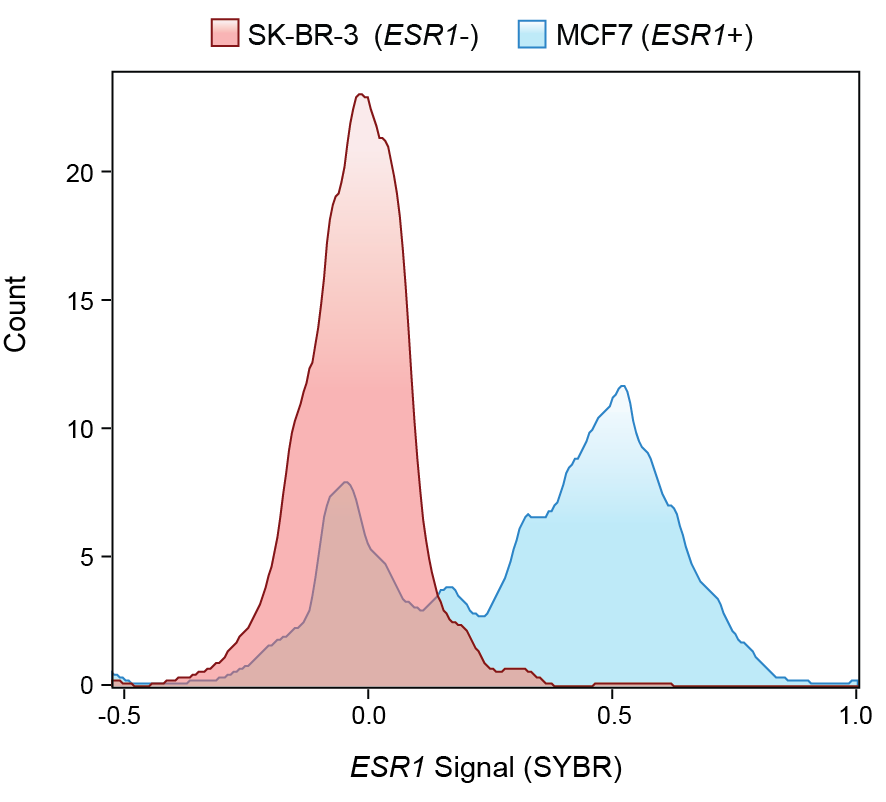
**

**Supplementary Figure 2.** SNAPD analysis of *ESR1* expression in SK-BR-3 and MCF7 cells. ESR1 is not expressed in SK-BR-3 cells, and is known to display heterogeneous expression in MCF7 cells.

**
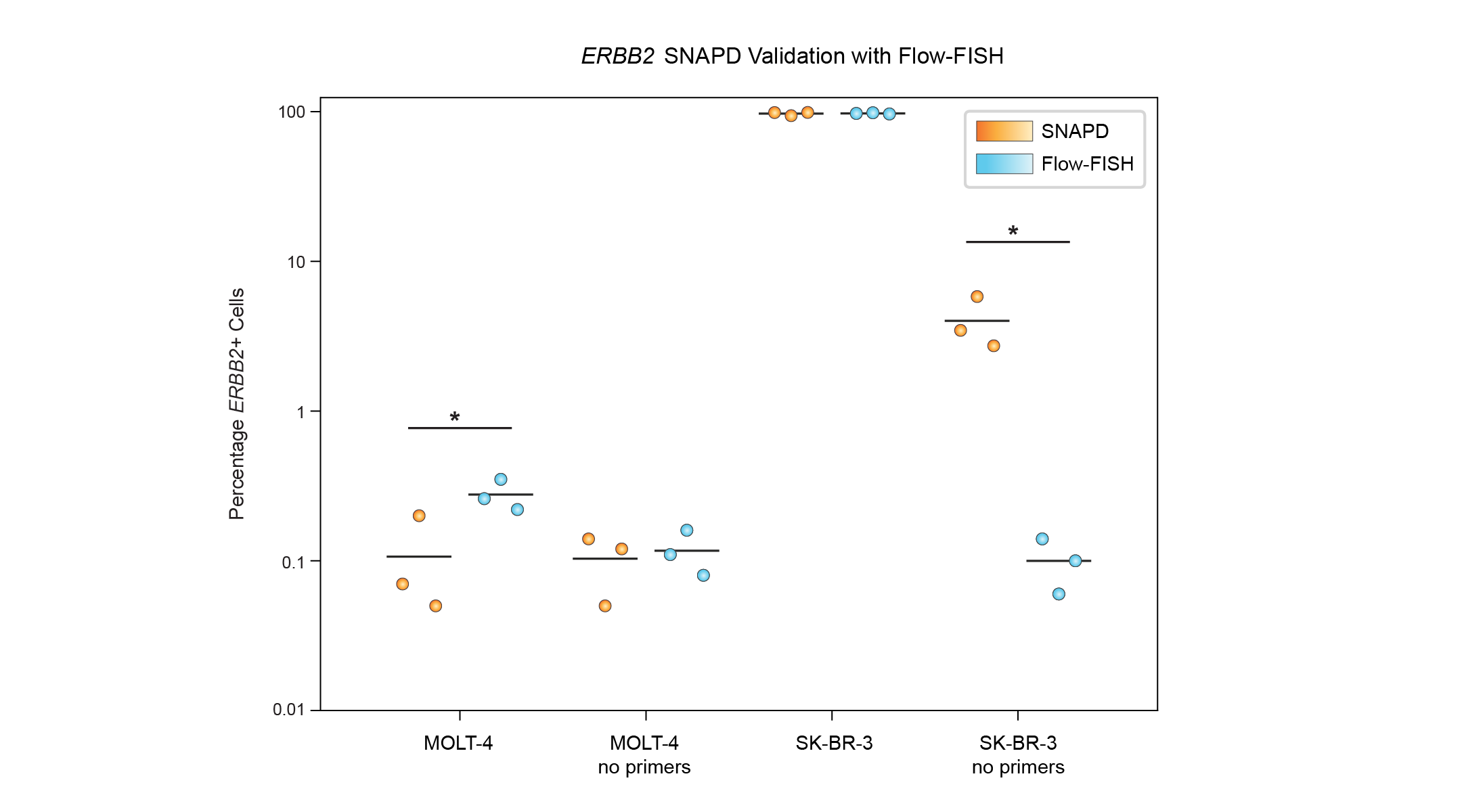
**

**Supplementary Figure 3.** *ERBB2* SNAPD and Flow-Fish comparison with no-primer controls. No statistically significant difference was observed in SK-BR-3 amplification between the two methods (p=0.895), although SNAPD showed slightly lower MOLT-4 amplification (p=0.049). SNAPD also showed some background signal in SK-BR-3 no primer controls, which is presumably due to autofluorescence or direct excitation of cell nuclei by the lasers. * p<0.05.

**
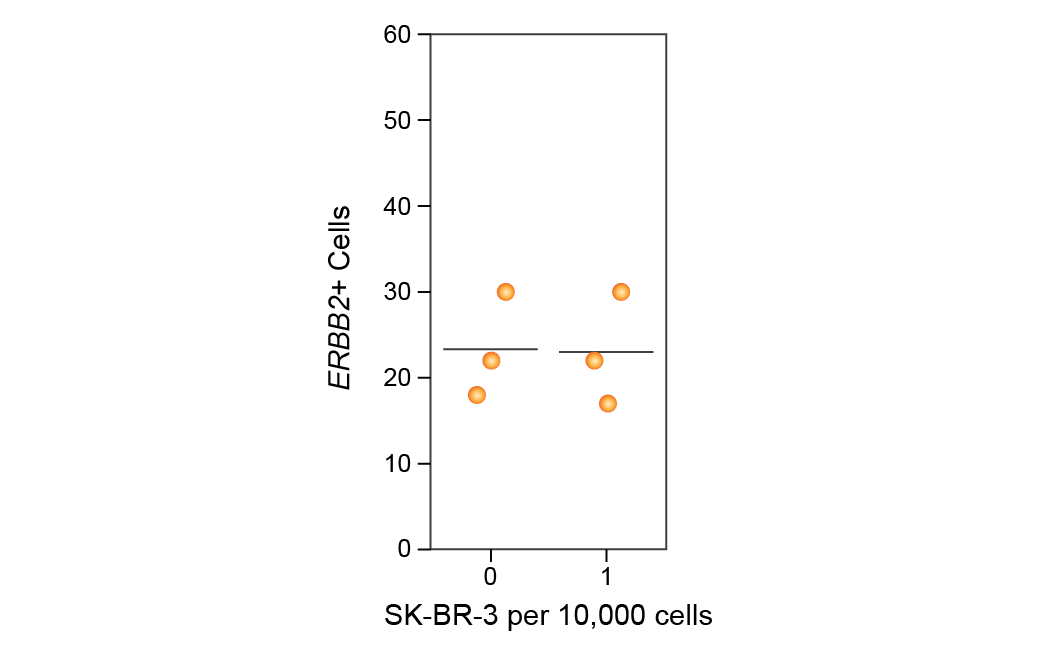
**

**Supplementary Figure 4.** *ERBB2* SNAPD limit of detection assays. SK-BR-3 cells at a prevalence of 1 in 10,000 total cells analyzed could not be distinguished from the negative control (p>0.05).


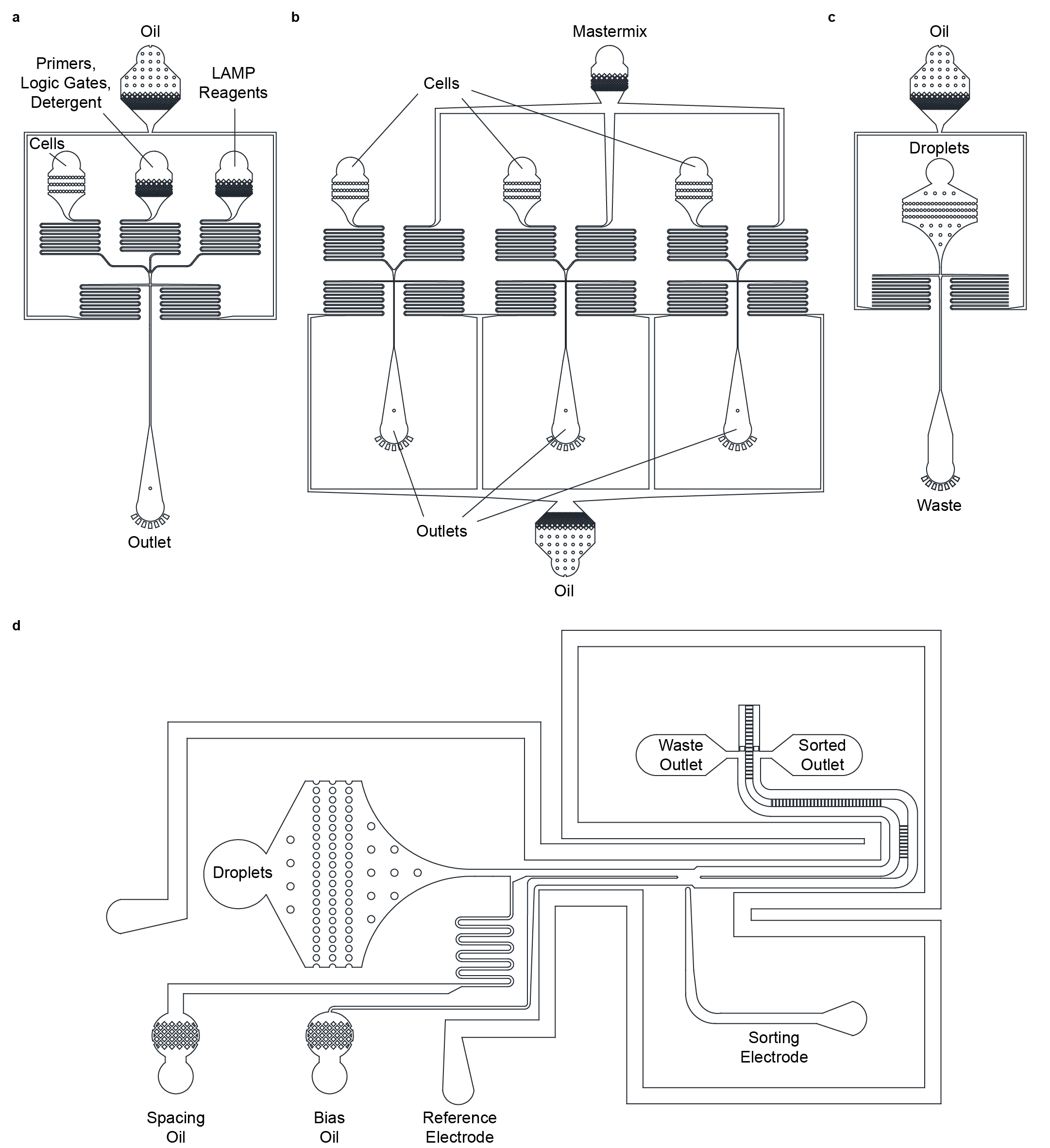


**Supplementary Figure 5.** Schematics for microfluidic designs. (**a**) Droplet generator with three inlets. The droplet junction is formed by the intersection of two orthogonal 60 um channels. (**b**) Triplicate droplet generator with two inlets that allows three different cell mixtures to be encapsulated in parallel. The droplet junction is formed by the intersection of two orthogonal 60 um channels. (**c**) Droplet analysis device. The droplet reinjection junction is formed by the intersection of two orthogonal 60 um channels.

(**d**) Droplet sorting device. The reinjection channel is 60 um in diameter. All devices were fabricated with a target channel height of 60 um.

**
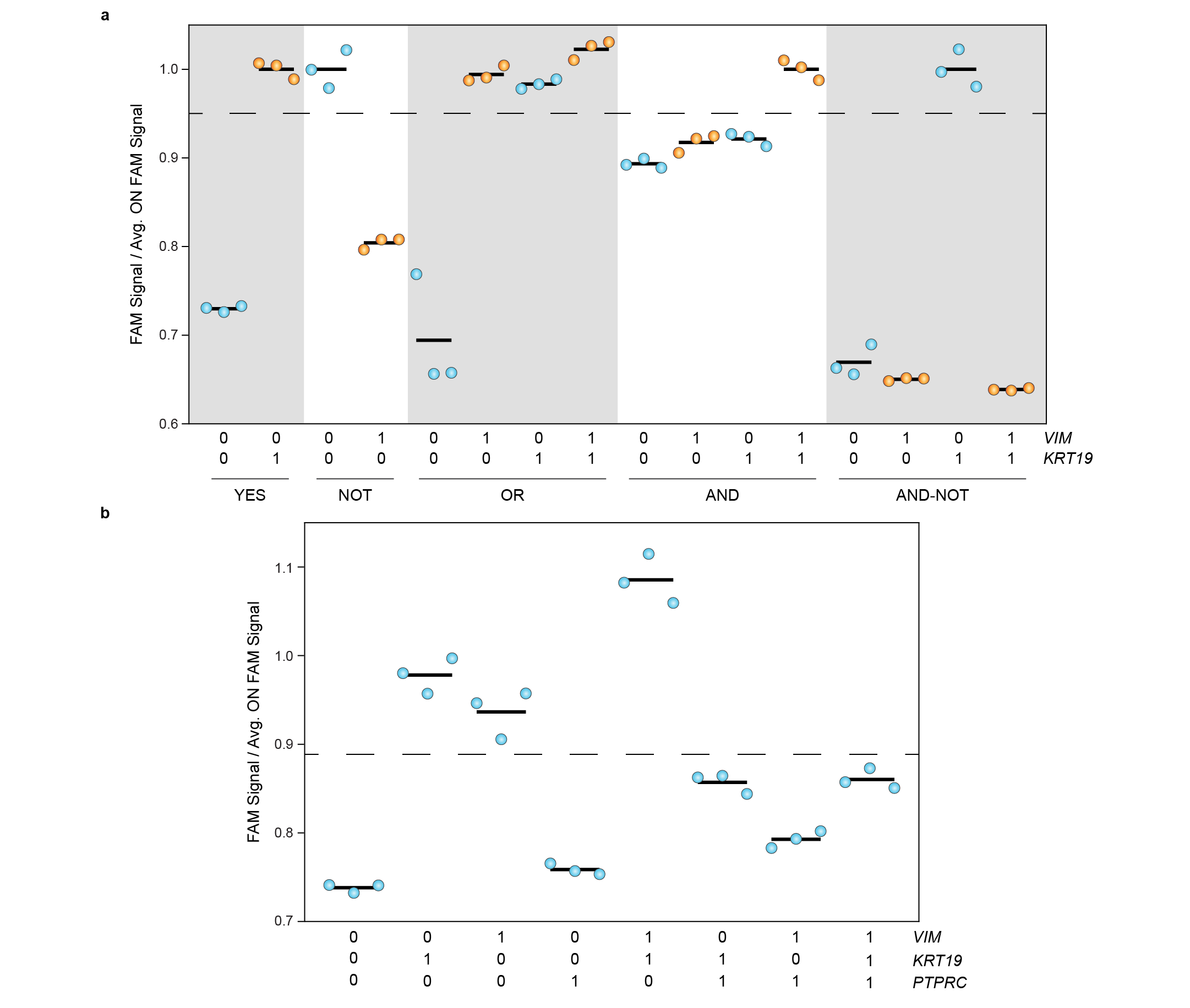
**

**Supplementary Figure 6.** Logic gate outputs in response to RNA inputs. Plots show an alternate representation of the data in Figs. 4a-f. Here, the raw FAM signal is divided by the raw FAM signal for the average ON state. (**a**) Data for YES, NOT, OR, AND, and AND-NOT logic gates shown in Figs. 4a-e. (**b**) Data for the logic circuit shown in Fig. 4f. All experiments were performed in triplicate.

**
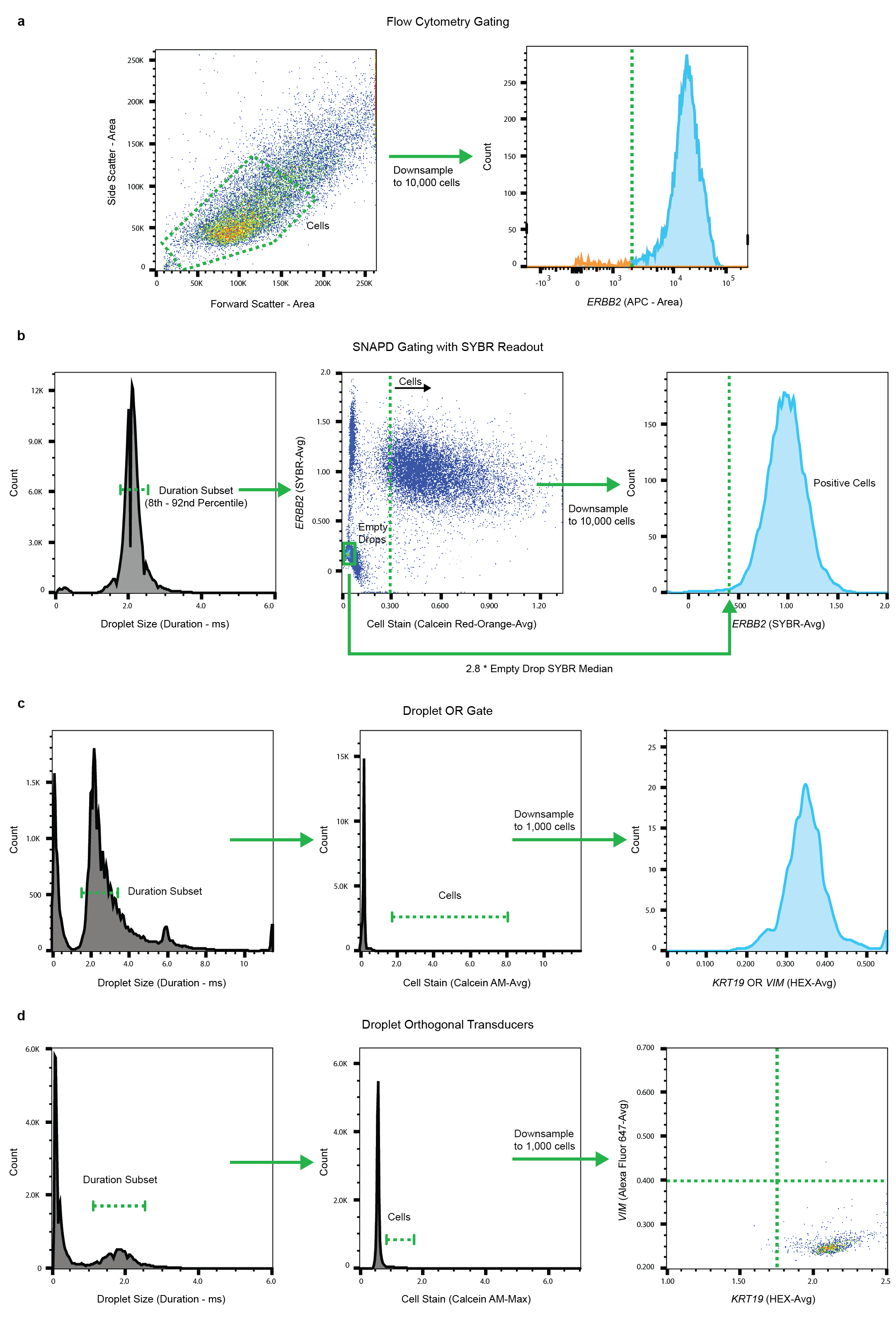
**

**Supplementary Figure 7.** Gating strategies for (**a**) flow cytometry, (**b**) single-target SNAPD with SYBR readout, (**c**) droplet OR gate, and (**d**) droplet orthogonal transducer experiments.

**
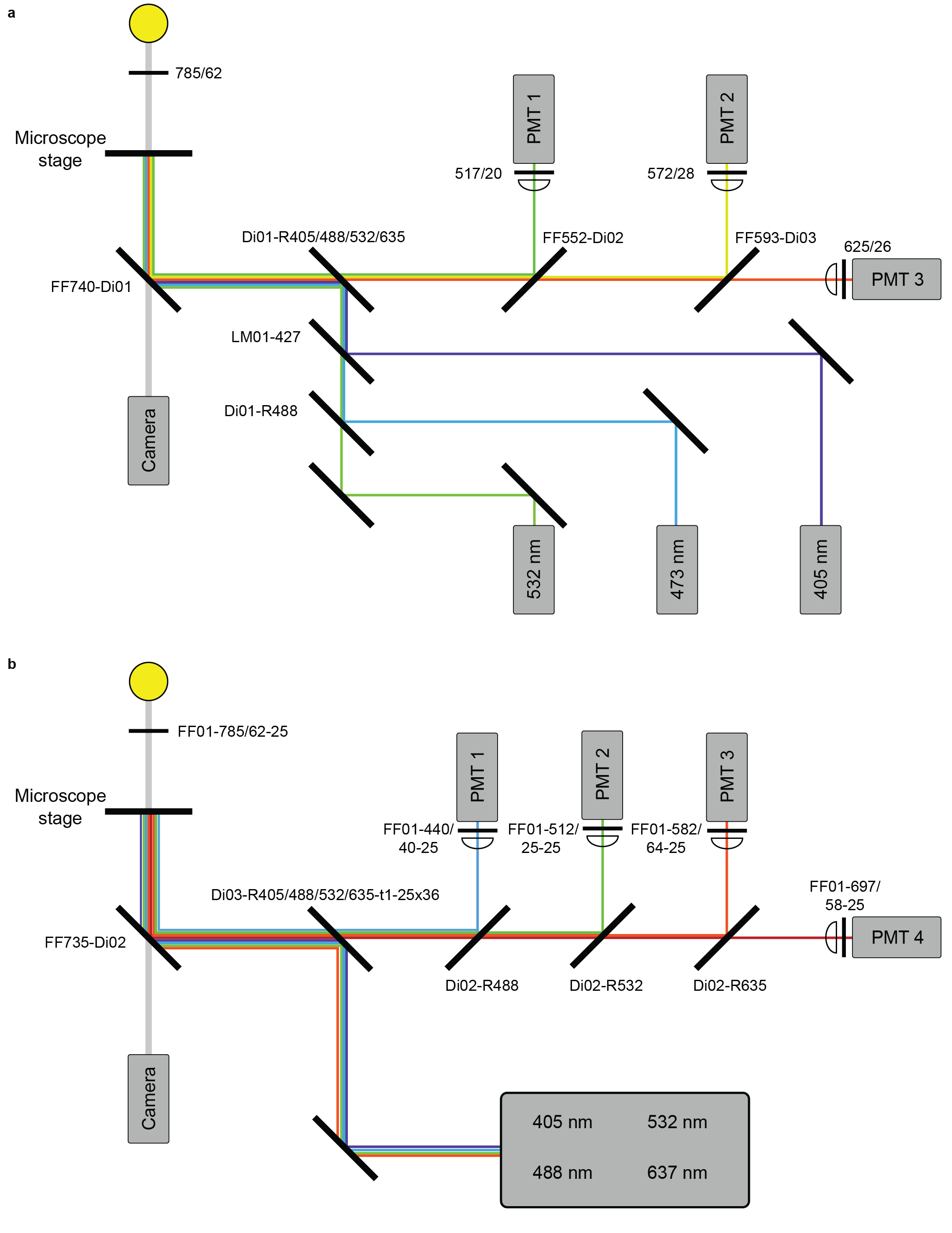
**

**Supplementary Figure 8.** Optical configurations used in this work. (**a**) The 3-channel optical configuration used for the majority of droplet experiments. All droplet data was collected using this configuration, with the exception of the droplet orthogonal transducer experiments in Figure 3c. (**b**) The 4-channel optical configuration used for the droplet orthogonal transducer experiments in Figure 3c of this paper.

**Supplementary Table 1.** DNA sequences of LAMP and qPCR primers, logic gates, and reporter complexes.

| ***KRT19* LAMP Primers** | | |
| --- | --- | --- |
| *KRT19* F3 | AGTGACATGCGAAGCCAAT | |
| *KRT19* B3 | GCTTTCATGCTCAGCTGTGA | |
| *KRT19* FIP | AGCGACCTCCCGGTTCAATTCTCGAGCAGAACCGGAAGGAT | |
| *KRT19* BIP | CACACGGAGCAGCTCCAGATGTGCAGCTCAATCTCAAGACC | |
| *KRT19* LF | TGGTGAACCAGGCTTCAGC | |
| *KRT19* LB | AGGTCCGAGGTTACTGACCTGC | |
|  | | |
| ***VIM* LAMP Primers** | | |
| *VIM* F3 | CCGCACCAACGAGAAGG | |
| *VIM* B3 | TGGTTAGCTGGTCCACCT | |
| *VIM* FIP | TCCAGGAAGCGCACCTTGTCGGAGCTGCAGGAGCTGAA | |
| *VIM* BIP | AAGATCCTGCTGGCCGAGCTCCCGCATCTCCTCCTCGTAG | |
| *VIM* LF | AGTTGGCGAAGCGGTCA | |
| *VIM* LB | CAGCTCAAGGGCCAAGGCAA | |
|  | | |
| ***RPL3* LAMP Primers** | | |
| *RPL3*-1 F3 | AAGGACTTCAGCAGCATGAA | |
| *RPL3*-1 B3 | CCGATGACGTCGATCATCTC | |
| *RPL3*-1 FIP | TTCTGGCGCAGAGGAAGCAGAAGTACTGCCAAGTCATCCG | |
| *RPL3*-1 BIP | CACTGTGGCCGAGAAGCTGGGCCCAAACACTTGGTTCACA | |
| *RPL3*-1 LF | TGGGTGTGGGCAATGACA | |
| *RPL3*-1 LB | GCGAGAGGCTTGAGCAGCA | |
| *RPL3*-2 F3 | CGTGGGAAGGTGAAGAGC | |
| *RPL3*-2 B3 | CCCACAATGCCCACAACC | |
| *RPL3*-2 FIP | ATGTGAGTCATGCCAGCCTTGTCCTAAGGATGACCCGTCCA | |
| *RPL3*-2 BIP | GGAAGTCGACAGGCCGGGATGGGTGGTGTCTCTACAATGG | |
| *RPL3*-2 LB | AGGTGAACAAGAAGGAGGTGG | |
|  | | |
| ***ERBB2* LAMP Primers** | | |
| *ERBB2*-1 F3 | CGGGGCCCTTTACTGCG | |
| *ERBB2*-1 B3 | CCTGGCAGCCCTGGTAG | |
| *ERBB2*-1 FIP | TGGCTCCGGCTGGACCCCCCCCACCCCTCGCA | |
| *ERBB2*-1 BIP | CCAAGTGTGCACCGGCACAGAGGTGGCGGAGCATGT | |
| *ERBB2*-1 LF | GCAAGAGGGCGAGGAGGA | |
| *ERBB2*-1 LB | TGAAGCTGCGGCTCCCT | |
| *ERBB2*-2 F3 | CCGGAGCCGCAGTGAG | |
| *ERBB2*-2 B3 | GCGATGAGCACGTAGCC | |
| *ERBB2*-2 FIP | TGCTCGCGGCTCCGGGGGAGCTGGCGGCCTTG | |
| *ERBB2*-2 BIP | GCCAGGTGGTGCAGGGAATGGATATCCTGCAGGAAGGA | |
| *ERBB2*-2 LB | GAACTCACCTACCTGCCCA | |
| ***PTPRC* LAMP Primers** | | |
| *PTPRC*-1 F3 | CCTTACAGTATTTTTGGAGAAGTT | |
| *PTPRC*-1 B3 | AGTTTAAGCCACAAATACATGG | |
| *PTPRC*-1 FIP | TGTTACCCTAAGAACAAACCACTTGACCGAATCTGACATCATCA | |
| *PTPRC*-1 BIP | GGAGGAAATTGTTCCTCGTCTGATTATCTGGAAGTCAGCCGT | |
| *PTPRC*-1 LF | CTAGCTGCATGAACTGCTAGG | |
| *PTPRC*-1 LB | AAGACAACAGTGGAGAAAGGACGC | |
| *PTPRC*-2 F3 | GGGTAACAGAGGAGGAAAT | |
| *PTPRC*-2 B3 | GGAAGGTGTTGGGCTTTG | |
| *PTPRC*-2 FIP | CCGTGTCCCTAAGAAACAGCGTTCCTCGTCTGATAAGACA | |
| *PTPRC*-2 BIP | CCAGATATGACCATGTATTTGTGGCTCACAAATACTTCTGTGTCCAG | |
| *PTPRC*-2 LF | TGCGTCCTTTCTCCACTGT | |
| *PTPRC*-2 LB | TAAACTCTTGGCATTTGGCTTTGC | |
|  |  | |
| **qPCR Primers** | | |
| *KRT19* qPCR F | ATAAAAGGCGCCAGGTGAGG | |
| *KRT19* qPCR R | CTTCAGTCCGGCTGGTGAAC | |
| *GAPDH* qPCR F | GTCTCCTCTGACTTCAACAGCG | |
| *GAPDH* qPCR R | ACCACCCTGTTGCTGTAGCCAA | |
|  | | |
| **Logic Strands** | | |
| *KRT19* Transducer 1 | CGAGTGCTGCGTATGACAAGGGCTAGCGTTATGCTACGAGCGACCTCCCGGTTCAATTCT | |
| Transducer 1 Output | AACGCTAGCCCTTGTCATACGCAGCACTCG | |
| *KRT19* Transducer 2 | CGCGATCCGAGTGCTGCGTATGACAAGGGCTAGCGTTTGCCGGAAGCGACCTCCCGGTTC | |
| *VIM* Transducer 2 | CGCGATCCGAGTGCTGCGTATGACAAGGGCTAGCGTTTGCCGGATCCAGGAAGCGCACCT | |
| Transducer 2 Output | TCCGGCAAACGCTAGCCCTTGTCATACGCAGCACTCGGATCGCG | |
| *KRT19* Transducer 3 | CTGCTCTCACGGAGGCGCACCGGTAAGGGTCATCGATGAGCGACCTCCCGGTTCAATTCT | |
| *VIM* Transducer 3 | CTGCTCTCACGGAGGCGCACCGGTAAGGGTCATCGATGTCCAGGAAGCGCACCTTGTC | |
| Transducer 3 Output | CGATGACCCTTACCGGTGCGCCTCCGTGAGAGCAG | |
| AND Transducer | CGAGTGCTGCGTATGACAAGGGCTAGCGTTATGCTGCTCTCACGG | |
| AND Output | AACGCTAGCCCTTGTCATACGCAGCACTCG | |
| AND Threshold | CCGCTGGTGATCACTCTGCTCTCACGGAGGCGCACCGGTAAGGGTCATCG | |
| *VIM* Transducer 4 | CCATCGCGGAGACACGGACATCGTTAAGGCAGCCTGTAGGCAGCCTCCAGGAAGCGCACC | |
| *KRT19* Transducer 4 | GTGTCTCCGCGATGGCGAGTGCTGCGTATGACAAGGGCTAGCGTTAGCGACCTCCCGGTT | |
| AND-NOT Inhibitor | GGCTGCCTACAGGCTGCCTTAACGATGTCCGTGTCTCCGCGATGG | |
| AND-NOT Output | AACGCTAGCCCTTGTCATACGCAGCACTCGCCATCGCGGAGACAC | |
| Transducer 5 Output | GCGGCAGTCAGTCAGTTAGATCAGGACGCGGCGGCG | |
| *VIM* Transducer 5 | CGCCGCCGCGTCCTGATCTAACTGACTGACTGCCGCTCCAGGAAGCGCACCTTGTC | |
| Transducer 6 Output | GCGAACGCTAGCCCTTGTCATACGCAGCACTCGGCG | |
| *KRT19* Transducer 6 | CGCCGAGTGCTGCGTATGACAAGGGCTAGCGTTCGCAGCGACCTCCCGGTTCAATTCT | |
| *VIM* Transducer 7 | GTGTCTCCGCGATGGCGAGTGCTGCGTATGACAAGGGCTAGCGTTTCCAGGAAGCGCACC | |
| *PTPRC* Transducer 8 | CCATCGCGGAGACACGGACATCGTTAAGGCAGCCTGTAGGCAGCCTGTTACCCTAAGAAC | |
| *PTPRC* Transducer 9 | CCATCGCGGAGACACAAGGCAGCCTGTAGGCAGCCTGTTACCCTAAGAACAAACCACTTG | |
| Transducer 9 Output | GGCTGCCTACAGGCTGCCTTGTGTCTCCGCGATGG | |
|  | | |
| **Fluorophore/Quencher Strands** | | |
| RepF-FAM* | **6-FAM**-CGAGTGCTGCGTATGACAAGGGCTAGCGTT | |
| RepF-HEX* | **HEX**-CGAGTGCTGCGTATGACAAGGGCTAGCGTT | |
| RepQ-RG* | CCCTTGTCATACGCAGCACTCG-**IowaBlackFQ** | |
| RepF2-AF647 | **AlexaFluor647**-CGCCGCGTCCTGATCTAACTGACTGACTGC | |
| RepQ2-FR | TCAGTTAGATCAGGACGCGGCG-**IowaBlackRQ** | |
| RepF2-FAM | **6-FAM**-CGCCGCGTCCTGATCTAACTGACTGACTGC | |
| RepQ2-RG | TCAGTTAGATCAGGACGCGGCG-**IowaBlackFQ** | |
|  |  | |
| ***In Vitro* Transcribed RNA Fragments** | | |
| Gene | NCBI Accession Number | Range (Bases) |
| *KRT19* | NM_002276.4 | 1 - 1470 |
| *VIM* | NM_003380.5 | 340 - 1019 |
| *PTPRC* | NM_080921.3 | 1 - 312 |

* Sequences adapted from Jiang et al.^1^

**Supplementary Table 2.** Strand concentrations used in logic experiments.

| **Experiment** | **Logic Gate and Reporter Concentrations** | **RNA Input Conc.** |
| --- | --- | --- |
| YES | 400 nM *KRT19* Transducer 1 : 200 nM Transducer 1 Output | 10 nM |
|  | 100 nM RepF-FAM : 200 nM RepQ-RG |  |
| NOT | 200 nM AND-NOT Output : 100 nM RepF-FAM | 1 nM |
|  | 240 nM *VIM* Transducer 4 : 220 nM AND-NOT Inhibitor |  |
|  | 200 nM RepQ-RG |  |
| AND | 120 nM *KRT19* Transducer 3 : 60 nM Transducer 3 Output | 10 nM each |
|  | 120 nM *VIM* Transducer 3 : 60 nM Transducer 3 Output |  |
|  | 55 nM AND Output : 60 nM AND Transducer |  |
|  | 65 nM AND Threshold |  |
|  | 50 nM RepF-FAM : 100 nM RepQ-RG |  |
| OR | 220 nM *KRT19* Transducer 2 : 200 nM Transducer 2 Output | 1 nM each |
|  | 220 nM *VIM* Transducer 2 : 200 nM Transducer 2 Output |  |
|  | 100 nM RepF-FAM : 200 nM RepQ-RG |  |
| AND-NOT | 220 nM *KRT19* Transducer 4 : 200 nM AND-NOT Output | 1 nM each |
|  | 240 nM *VIM* Transducer 4 : 220 nM AND-NOT Inhibitor |  |
|  | 100 nM RepF-FAM : 200 nM RepQ-RG |  |
| Circuit | 110 nM *KRT19* Transducer 4 : 100 nM AND-NOT Output | 1 nM each |
|  | 110 nM *VIM* Transducer 7 : 100 nM AND-NOT Output |  |
|  | 220 nM *PTPRC* Transducer 8 : 200 nM AND-NOT Inhibitor |  |
|  | 220 nM *PTPRC* Transducer 9 : 200 nM Transducer 8 Output |  |
|  | 100 nM RepF-FAM : 200 nM RepQ-RG |  |
| 2 Transducers (lysate) | 220 nM *VIM* Transducer 5 : 200 nM Transducer 5 Output | - |
|  | 220 nM *KRT19* Transducer 6 : 200 nM Transducer 6 Output |  |
|  | 100 nM RepF2-FAM : 200 nM RepQ2-RG |  |
|  | 100 nM RepF-HEX : 200 nM RepQ-RG |  |
| OR (droplets) | 110 nM *KRT19* Transducer 3 : 100 nM Transducer 3 Output | - |
|  | 110 nM *VIM* Transducer 3 : 100 nM Transducer 3 Output |  |
|  | 100 nM RepF-HEX : 200 nM RepQ-RG |  |
| 2 Transducers (droplets) | 110 nM *KRT19* Transducer 2 : 100 nM Transducer 2 Output | - |
|  | 110 nM *VIM* Transducer 5 : 100 nM Transducer 5 Output |  |
|  | 100 nM RepF-HEX : 200 nM RepQ-RG |  |
|  | 100 nM RepF2-AF647 : 200 nM RepQ2-FR |  |

**Supplementary Table 3.** Solutions and flowrates used in microfluidic experiments.

|  | **Flow Rate (µL/hr)** | **Composition** |
| --- | --- | --- |
|  | | |
| **2-Inlet drop generator device** | | |
| Inlet 1 | 80 | 10X cell suspension in Fluorobrite™ DMEM (Gibco) with 12.4 U/µL RNase If (New England Biolabs) and 0.025 U/µL DNase I (Thermo Fisher) |
| Inlet 2 | 720 | WarmStart 2.0 LAMP Master Mix with included indicator dye (New England Biolabs), 2.78% Tween-20 (Sigma Aldrich), 1.38U/µL RNasin Plus RNase Inhibitor (Promega), 0.025 U/µL DNase I (Thermo Fisher), 55.56mM Dithiothreitol (DTT), and LAMP Primers |
| Oil | 1,600 | Bio Rad Droplet Digital PCR oil |
|  | | |
| **3-Inlet drop generator device** | | |
| Inlet 1 | 80 | 10X cell suspension in DPBS (Gibco) |
| Inlet 2 | 400 | WarmStart 2.0 LAMP Master Mix (New England Biolabs) |
| Inlet 3 | 320 | LAMP primers, DNA complexes, 1.25U/µL SUPERase• In™ RNase Inhibitor (Intitrogen), LAMP indicator dye (New England Biolabs), 6.25% Tween-20 (Sigma Aldrich) |
| Oil | 1,200 | Bio Rad Droplet Digital PCR oil |
|  |  |  |
| **Droplet analysis device** | | |
| Inlet 1 | 400 | SNAPD droplets |
| Oil | 800 | Bio Rad Droplet Digital PCR oil |
|  |  |  |
| **Droplet sorting device** | | |
| Inlet 1 | 100 | SNAPD droplets |
| Spacing Oil | 400 | Bio Rad Droplet Digital PCR oil |
| Bias Oil | 1,000 | Bio Rad Droplet Digital PCR oil |
| Inlet 2 | 0 | 1 M NaCl (Sorting Electrode) |
| Inlet 3 | 0 | 1 M NaCl (Reference Electrode) |

**References**

1. Jiang, Y. S., Li, B., Milligan, J. N., Bhadra, S. & Ellington, A. D. Real-Time Detection of Isothermal Amplification Reactions with Thermostable Catalytic Hairpin Assembly. *JACS* 7430–7433 (2013).
